# Source and Purity of Dengue-Viral Preparations Impact Requirement for Enhancing Antibody to Induce Elevated IL-1β Secretion: A Primary Human Monocyte Model

**DOI:** 10.1371/journal.pone.0136708

**Published:** 2015-08-24

**Authors:** Justin B. Callaway, Scott A. Smith, Douglas G. Widman, Karen P. McKinnon, Frank Scholle, Gregory D. Sempowski, Dirk P. Dittmer, James E. Crowe, Aravinda M. de Silva, Jenny P.-Y. Ting

**Affiliations:** 1 Department of Microbiology and Immunology, The University of North Carolina at Chapel Hill, Chapel Hill, NC, United States of America; 2 The Lineberger Comprehensive Cancer Center, The University of North Carolina at Chapel Hill, Chapel Hill, NC, United States of America; 3 The Vanderbilt Vaccine Center, Vanderbilt Medical Center, Nashville, TN, United States of America; 4 Department of Medicine, Vanderbilt Medical Center, Nashville, TN, United States of America; 5 Department of Biological Sciences, North Carolina State University, Raleigh, NC, United States of America; 6 Duke Human Vaccine Institute, Durham, NC, United States of America; 7 Department of Pathology, Microbiology and Immunology, Vanderbilt Medical Center, Nashville, TN, United States of America; 8 Department of Pediatrics, Vanderbilt Medical Center, Nashville, TN, United States of America; 9 Department of Genetics, The University of North Carolina at Chapel Hill, Chapel Hill, NC, United States of America; University of Rochester, UNITED STATES

## Abstract

Dengue virus is a major global health threat and can lead to life-threatening hemorrhagic complications due to immune activation and cytokine production. Cross-reactive antibodies to an earlier dengue virus infection are a recognized risk factor for severe disease. These antibodies bind heterologous dengue serotypes and enhance infection into Fc-receptor-bearing cells, a process known as antibody-dependent enhancement of infection. One crucial cytokine seen elevated in severe dengue patients is IL-1β, a potent inflammatory cytokine matured by the inflammasome. We used a highly-physiologic system by studying antibody-dependent enhancement of IL-1β in primary human monocytes with anti-dengue human monoclonal antibodies isolated from patients. Antibody-enhancement increased viral replication in primary human monocytes inoculated with supernatant harvested from Vero cells infected with dengue virus serotype 2 (DENV-2) 16681. Surprisingly, IL-1β secretion induced by infectious supernatant harvested from two independent Vero cell lines was not enhanced by antibody. Secretion of multiple other inflammatory cytokines was also independent of antibody signaling. However, IL-1β secretion did require NLRP3 and caspase-1 activity. Immunodepletion of dengue virions from the infectious supernatant confirmed that virus was not the main IL-1β-inducing agent, suggesting that a supernatant component(s) not associated with the virion induced IL-1β production. We excluded RNA, DNA, contaminating LPS, viral NS1 protein, complement, and cytokines. In contrast, purified Vero-derived DENV-2 16681 exhibited antibody-enhancement of both infection and IL-1β induction. Furthermore, C6/36 mosquito cells did not produce such an inflammatory component, as crude supernatant harvested from insect cells infected with DENV-2 16681 induced antibody-dependent IL-1β secretion. This study indicates that Vero cells infected with DENV-2 16681 may produce inflammatory components during dengue virus propagation that mask the virus-specific immune response. Thus, the choice of host cell and viral purity should be carefully considered, while insect-derived virus represents a system that elicits antibody-dependent cytokine responses to dengue virus with fewer confounding issues.

## Introduction

With an estimated 390 million global infections per year, dengue virus (DENV) is the most burdensome arbovirus in the world [1]. The four distinct serotypes (DENV-1–DENV-4) are transmitted by the widespread, tropical *Aedes aegypti* and *Aedes albopictus* mosquitoes, and nearly half of the global population lives in DENV-endemic regions [2]. A first infection with any serotype may cause an asymptomatic infection or a mild to severe flulike illness referred to as dengue fever (DF) [3]. Patients typically recover without complication and develop long-term immunity to the same DENV serotype, but immunity to heterologous serotypes is transient [4, 5]. Upon later infection with a second serotype of DENV, a small percentage of patients progress to the life-threatening disease course of severe dengue [2, 4]. During severe dengue, a reversible permeability develops in the vasculature, causing hemorrhagic manifestations and potential hypovolemic shock [3, 6]. There is no specific cure or vaccine, but supportive therapy until the disease course passes can reduce mortality levels from greater than 20% to less than 1% [2, 7].

It is now widely accepted that cross-reactive antibodies to a primary infection can increase disease severity during a heterologous DENV infection [8]. These antibodies may enhance infection of DENV into Fc-receptor-bearing cells by a mechanism known as antibody-dependent enhancement (ADE) of infection [3, 9]. Circulating CD14^+^ monocytes, which express high levels of Fc receptors, have been identified as the primary target of ADE among all peripheral blood mononuclear cells (PBMCs), and increased activation of monocytes is associated with more severe dengue disease [10, 11].

It is believed that a “cytokine storm,” a massive and aberrant upregulation of cytokine production, contributes to vascular permeability and hemorrhagic complications [3, 7]. A substantial number of patient studies have identified the upregulation of a wide array of cytokines during DENV infection [7, 12–17]. The lack of a consensus on the most damaging cytokines likely reflects the complicated nature of defining disease severity and achieving consistency between study parameters. As well, abundant evidence identifies the importance of the specific sequence of infecting serotypes, with a primary DENV-1 infection followed by a secondary DENV-2 infection carrying a much higher risk of severe disease development than other combinations [3].

One inflammatory cytokine that is elevated in many cytokine profiles of DENV patients is IL-1β. Higher levels of circulating IL-1β have been detected in the sera of severe dengue patients compared to DF patients [12, 18]. Also, 10-fold higher expression of the gene *IL1B*, which encodes the pro-IL-1β zymogen, has been measured in PBMCs of severe dengue patients compared to DF patients [15, 18]. Further, monocytes infected in culture with DENV have been found to secrete IL-1β [19, 20]. As IL-1β is a potent inflammatory cytokine, its mechanism of production during ADE may provide insight into severe disease pathogenesis.

The unique regulation of IL-1β production and secretion is generally controlled by a two-step process. The first step is transcriptional and translational stimulation (such as via NF-κB activation downstream of TLR signaling) leading to increased expression of *IL1B* and translation of the pro-IL-1β zymogen [21]. Alone, pro-IL-1β expression is not sufficient to induce secretion of active IL-1β. Instead, the second step of the control mechanism is the activation of the inflammasome [22]. The inflammasome is a large, multi-protein complex typically containing one or more nucleotide-binding domain, leucine-rich repeat containing (NLR) proteins which, upon assembly, recruit in multiple copies of the pro-caspase-1 zymogen [23–25]. Autocatalytic cleavage occurs when pro-caspase-1 proteins come into close association with one another, forming active caspase-1 [24, 26, 27]. Active caspase-1 subsequently cleaves pro-IL-1β and pro-IL-18 into mature IL-1β and IL-18 [22, 28]. More recent studies have revealed that other, non-NLR proteins, such as AIM2 and RIG-I can also form an inflammasome [29, 30]. The stimuli that activate the inflammasome vary by specific NLR component. To date, the NLRP3 inflammasome is recognized to respond to the widest variety of pathogen- and danger-associated molecular patterns [23]. Additionally, DENV has been reported to activate the NLRP3 inflammasome in polarized human macrophages [31].

We have recently shown that ADE of DENV harvested from infected C6/36 mosquito cells enhances IL-1β secretion by primary human monocytes in a mechanism dependent upon NLRP3 and caspase-1 [20]. However, DENV is also commonly propagated in Vero cells. Here, we employed a highly-relevant physiological system utilizing not only primary human monocytes, but also monoclonal antibodies (mAbs) isolated from DENV-immune patients [32, 33], to study ADE-induced IL-1β using DENV propagated in Vero cells. Interestingly, we found that inoculation with crude supernatant harvested from Vero cells infected with DENV-2 strain 16681 induced equivalent IL-1β secretion by primary monocytes in the presence or absence of enhancing antibodies. Further studies indicated that the secretion of IL-1β was completely independent of antibody signaling and viral replication. Removal of DENV-2 16681 virions from crude supernatant preparations indicated that a soluble component present in the supernatant was responsible for the induction of IL-1β. In contrast, purified Vero-derived DENV-2 16681 induced elevated IL-1β secretion only in the presence of ADE. Similarly, crude supernatant harvested from C6/36 mosquito cells infected with DENV-2 16681 induced ADE-dependent IL-1β secretion without the need to purify the viral preparation. Interestingly, crude supernatant from Vero cells infected with a second DENV strain induced ADE-dependent IL-1β. Collectively these data indicate that cell-line, strain, and purity selections during DENV preparation should be carefully considered before undertaking a study investigating DENV-induced cytokine production.

## Results

### Primary human monocytes are the main blood cell type infected by DENV

We first sought to confirm that CD14^+^ monocytes were the target cell of dengue virus (DENV) in the current system. Mobilized PBMCs were isolated from leukapheresed blood and inoculated with a multiplicity of infection (MOI) of 50 focus-forming units (ffu) of DENV-2 strain 16681 that had been incubated with 1 μg/ml anti-DENV prM human monoclonal antibody (mAb) 5G22. Importantly, infectious viral titer was calculated via immunoassay on Vero cells. Vero cells are highly susceptible to DENV infection, while monocytes are very resistant to the virus. Thus, an MOI of 50 does not directly translate to 50 times the amount of DENV needed to infect 1 monocyte. Crude culture supernatant harvested from DENV-infected Vero cells was cleared of cellular debris and used as the inoculum onto monocytes, hereafter referred to as DENV-Infectious Vero-cell (DIV) crude supernatant. The human mAb 5G22, isolated from a DENV-immune patient, is known to potently enhance DENV infection [32].

**Fig 1:**
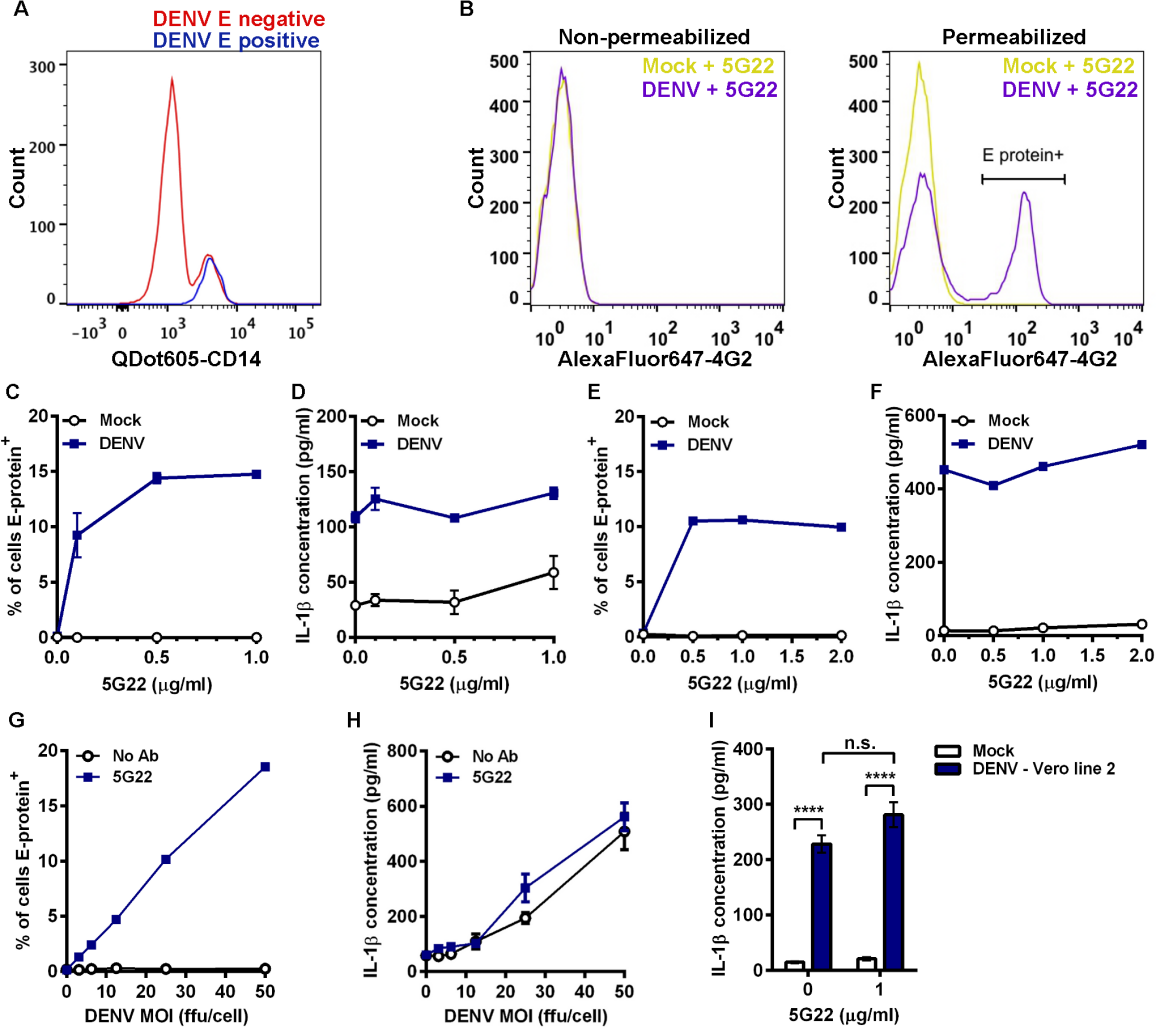
DIV crude supernatant induces IL-1β secretion independent of ADE. (A) Flow-cytometric histogram overlay comparing CD14 expression levels in cells negative or positive for intracellular DENV E protein at 24 hpi. (B) Mobilized monocytes were inoculated with mock medium or DIV crude supernatant that had been incubated with 1 μg/ml mAb 5G22. At 1 hpi, cells were washed and resuspended in fresh medium. At 24 hpi, cells were washed and stained for surface expression (left panel) or intracellular expression (right panel) of DENV E protein with mAb 4G2 conjugated to AlexaFluor 647 and analyzed by flow cytometry. (C) Cumulative percentages of mobilized monocytes positive for intracellular DENV E protein at 24 hpi with DIV crude supernatant in the presence of increasing concentrations of mAb 5G22. (D) Measurement of secreted IL-1β by ELISA using 24-hpi supernatants from 1C. (E) Repeat of 1C using fresh, non-mobilized monocytes (one value per point). (F) Measurement of secreted IL-1β using supernatants from 1E (one value per point). (G) Measurement of DENV E-protein expression in mobilized monocytes at 24 hpi. Cells were inoculated with increasing doses of DIV crude supernatant with or without 1 μg/ml mAb 5G22. (H) Measurement of secreted IL-1β using 24-hpi supernatants from 1G. (I) Secreted IL-1β by mobilized monocytes at 24 hpi with mock supernatant or DIV crude supernatant derived from a second line of Vero cells. For all figures: * = p < 0.05, ** = p < 0.01, *** = p < 0.001, **** = p < 0.0001, and “n.s.” = not significant (p > 0.05). Test used: Two-Way ANOVA with Tukey’s post-test (I).

At 24 hours post-inoculation (hpi), the inoculated PBMCs were analyzed by flow cytometry for both CD14 and DENV E-protein expression (Fig 1A). Intracellular DENV E protein detected by flow cytometry is a measure of viral replication [34]. Cells negative for DENV E protein expressed varying levels of CD14, while all cells positive for intracellular DENV E protein also expressed high levels of CD14. These data confirm that CD14^+^ monocytes are the target of ADE in a mixed population of PBMCs. Thus, most of the subsequent experiments utilize cryopreserved CD14^+^ mobilized monocytes that were purified using negative isolation to prevent activation through CD14. The advantage of mobilized, leukapheresed blood is that it provided a large source of cells from a single blood draw (with two separate donors total), eliminating much of the variability associated with multiple blood draws from different volunteers over time. Key phenotypes were verified using CD14^+^ monocytes purified from fresh draws of non-mobilized blood.

To verify that this flow-based assay was not detecting DENV adsorbed to the cell surface, we stained purified mobilized monocytes in the presence or absence of membrane permeabilization (Fig 1B). Inoculation with DIV crude supernatant and mAb 5G22 did not induce elevated DENV E-protein expression in non-permeabilized cells (Fig 1B, left panel). However, inoculation with DIV crude supernatant and mAb 5G22 induced a shift in DENV E-protein expression in permeabilized cells (Fig 1B, right panel). These data confirm that this flow-based assay does not detect surface-adsorbed virus.

### DIV crude supernatant induces IL-1β secretion by primary monocytes independent of enhancement with mAb 5G22

We next examined the induction of IL-1β in primary monocytes by DIV crude supernatant in the context of ADE. In the absence of mAb 5G22, no intracellular expression of DENV E protein was detected at 24 hpi in mobilized monocytes (Fig 1C). In contrast, pre-incubation of DIV crude supernatant with increasing levels of mAb 5G22 caused a dose-dependent increase in intracellular E-protein expression. Unexpectedly, DIV crude supernatant induced IL-1β secretion by mobilized monocytes independent of the presence of mAb 5G22, as measured by ELISA on monocyte supernatants collected at 24 hpi (Fig 1D). An identical pattern was observed in freshly-isolated monocytes from a non-mobilized donor, with mAb 5G22 required for intracellular E-protein expression but not for induction of IL-1β secretion (Fig 1E and 1F). We next inoculated mobilized monocytes with varying doses of DIV crude supernatant in the presence of a constant antibody dose or control condition. Increasing doses of DIV crude supernatant increased intracellular E-protein expression in the presence of 1 μg/ml mAb 5G22 (Fig 1G). However, increasing doses of DIV crude supernatant induced elevated secretion of IL-1β regardless of the presence of mAb 5G22 (Fig 1H). These data indicate a bifurcation in the dependence on anti-DENV antibody, with mAb 5G22 enhancing DENV replication but being dispensable for IL-1β secretion in primary monocytes inoculated with DIV crude supernatant.

To rule out the possibility that these outcomes are unique to the Vero cell line we used, crude supernatant was harvested from a second, independent line of Vero cells infected with DENV-2 strain 16681. IL-1β secretion induced by DIV crude supernatant harvested from this second Vero line also was independent of the presence of mAb 5G22 (Fig 1I). In total, these results indicate that anti-DENV antibodies significantly impact viral replication in primary monocytes but are completely dispensable for IL-1β secretion induced by DIV crude supernatant.

### DIV crude supernatant induces broad inflammatory cytokine secretion independent of ADE

**Fig 2:**
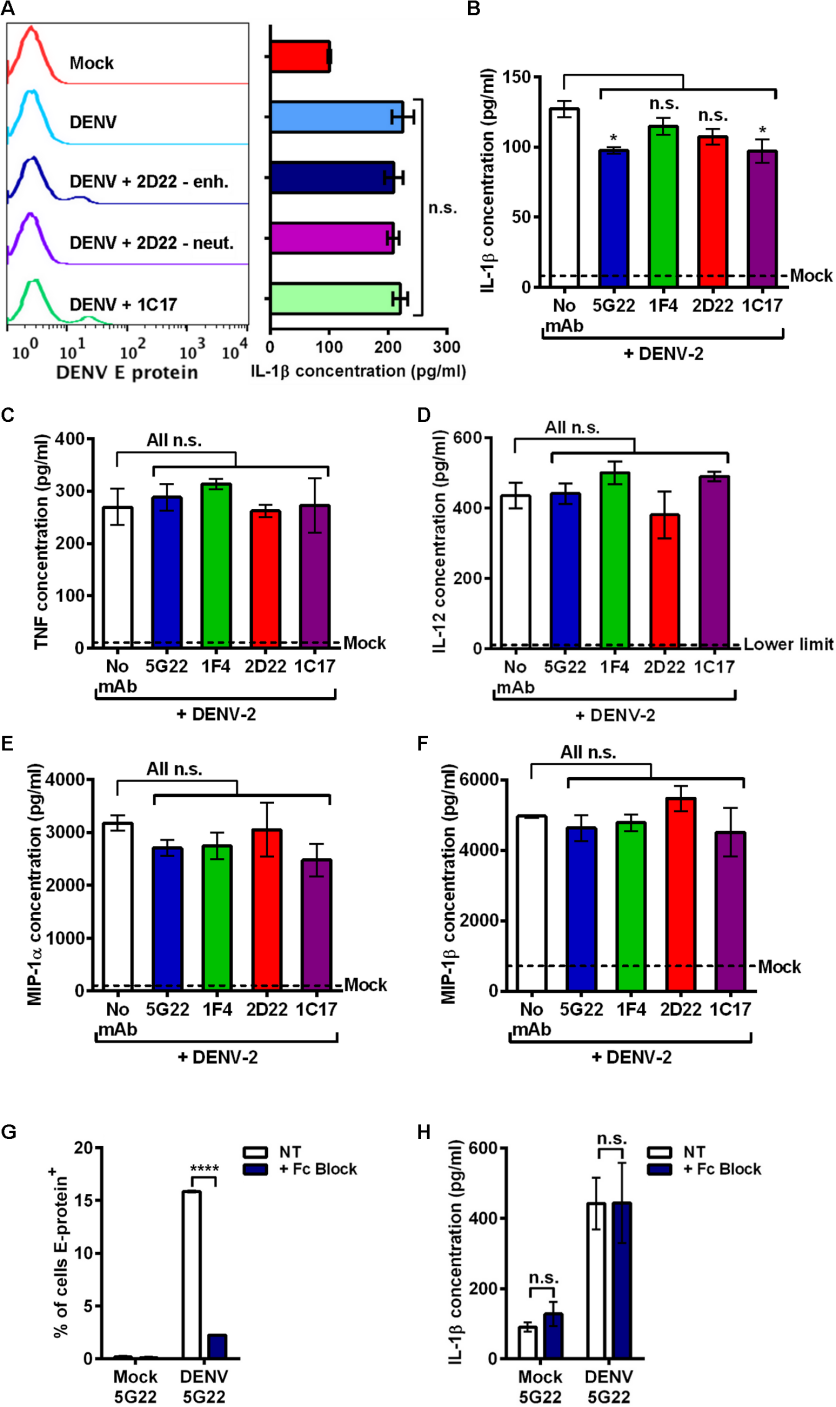
DIV crude supernatant induces inflammatory cytokine secretion independent of antibody signaling. (A) Left: Flow-cytometric histograms of 24-hpi DENV E-protein expression in mobilized monocytes after inoculation with mock supernatant, DIV crude supernatant alone, or DIV crude supernatant that was pre-incubated with 0.1 μg/ml mAb 2D22 (enhancing), 2 μg/ml mAb 2D22 (neutralizing), or 2 μg/ml mAb 1C17 (enhancing). Right: Measurement of secreted IL-1β at 24 hpi for corresponding samples. (B–F) Mobilized monocytes were inoculated with mock medium, DIV crude supernatant alone, or DIV crude supernatant that had been incubated with 0.1 μg/ml of mAbs 5G22, 1F4, 2D22, or 1C17. At 1 hpi, cells were washed to remove inoculum and resuspended in fresh medium. At 24 hpi, supernatants were collected, and cytokine secretion was evaluated by multiplex array, including inflammatory cytokines IL-1β (B), TNF (C), IL-12 (D), MIP-1α (E), and MIP-1β (F). Dashed lines indicate mean concentration induced by mock medium, except for panel D, in which mock-induced values fell below the lower limit of detection. (G) Measurement of intracellular DENV E-protein at 24 hpi in mobilized monocytes that were incubated with PBS or Fc-receptor binding inhibitor prior to inoculation with DIV crude supernatant. (H) Secreted IL-1β at 24 hpi using supernatants from 2G. Tests used: One-Way ANOVA (within DENV treatment) with Tukey’s post-test (A), One-Way ANOVA with Dunnett’s post-test (B–F), and Two-Way ANOVA with Bonferroni’s post-test (G and H).

To verify that the lack of ADE-induced IL-1β was not restricted to mAb 5G22, we tested additional antibody conditions with other human mAbs isolated from DENV-immune patients. Enhancement of DENV infection with 0.1 μg/ml mAb 2D22 and 2 μg/ml mAb 1C17 (indicated by peaks with higher DENV E-protein expression in the histograms) both failed to enhance IL-1β secretion induced by DIV crude supernatant (Fig 2A). In addition to enhancement of infection, mAb 2D22 also strongly neutralizes DENV-2 infection at higher concentrations, as previously reported [32]. The use of a neutralizing dose of mAb 2D22 at 2 μg/ml (indicated by the loss of elevated DENV E-protein expression compared to 0.1 μg/ml 2D22) also did not affect IL-1β secretion. These data confirm that DIV crude supernatant induces IL-1β secretion independent of antibody condition.

To assess if other cytokines are induced in a similar way, we inoculated mobilized monocytes with control medium, DIV crude supernatant alone, or DIV crude supernatant that had been incubated with 0.1 μg/ml of mAbs 5G22, 1F4, 2D22, or 1C17. Monoclonal antibody 1F4 is specific to DENV-1 and serves as an isotype-matched control antibody [33]. At 24 hpi, supernatants were collected and assayed for a number of inflammatory cytokines by multiplex array (Fig 2B–2F). As expected, antibody against DENV did not increase IL-1β secretion induced by DIV crude supernatant (Fig 2B). DIV crude supernatant also induced elevated secretion of inflammatory cytokines TNF (Fig 2C), IL-12 (Fig 2D), MIP-1α (Fig 2E), and MIP-1β (Fig 2F). Various antibodies against DENV did not enhance secretion of these cytokines.

We next antagonized antibody binding by pre-incubating mobilized monocytes with an Fc-receptor binding inhibitor. Fc-receptor inhibition significantly reduced intracellular, ADE-induced E-protein expression (Fig 2G) but did not affect the secretion of IL-1β (Fig 2H). In sum, these data confirm that DIV crude supernatant induces a number of inflammatory cytokines independent of antibody signaling.

### IL-1β secretion induced by DIV crude supernatant precedes viral replication

**Fig 3:**
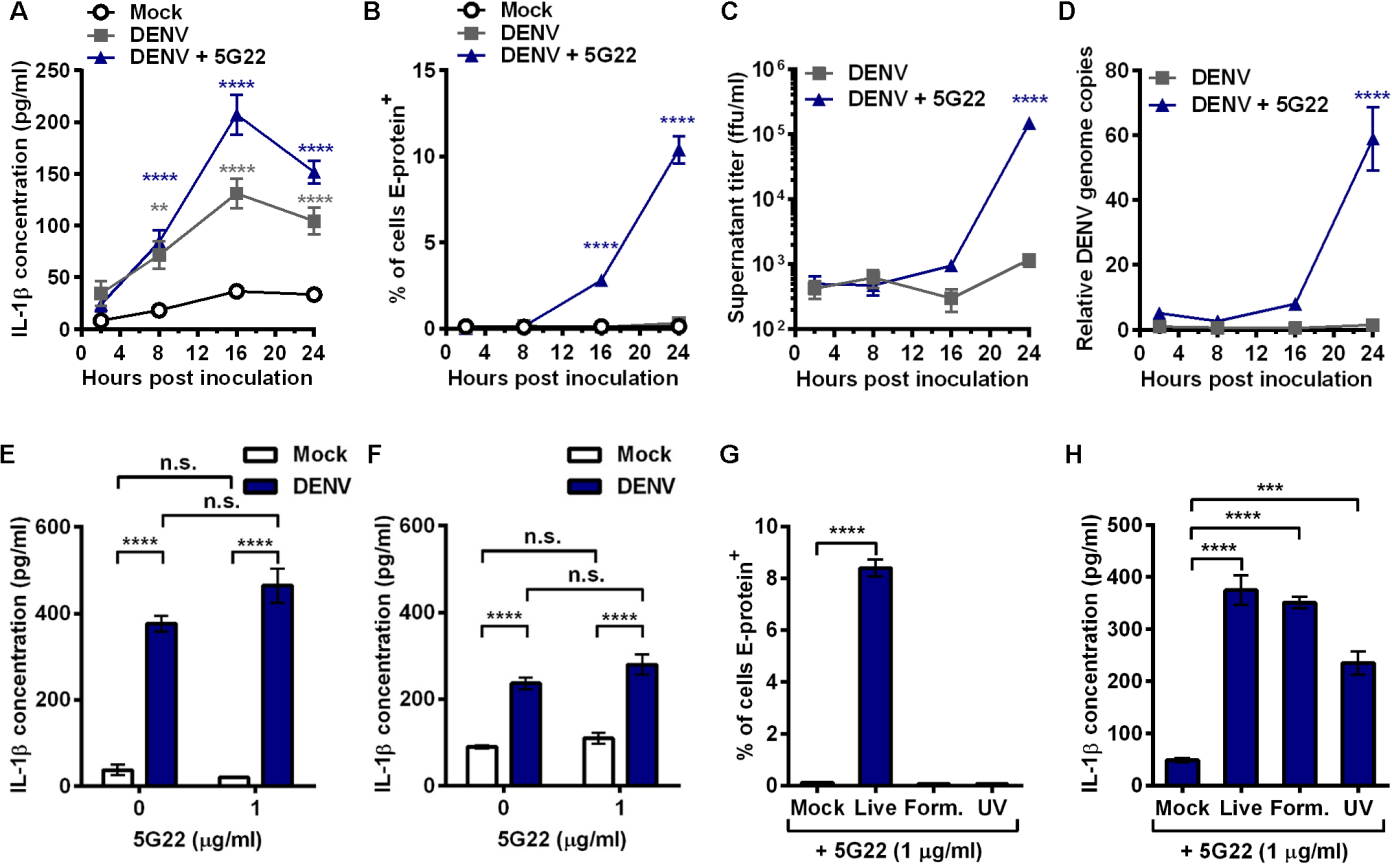
DIV crude supernatant induces rapid IL-1β secretion independent of viral replication. (A-D) Time course of mobilized monocytes inoculated with mock supernatant or DIV crude supernatant with or without mAb 5G22. Cells were washed at 1 hpi and resuspended in fresh medium. Samples were collected at 2, 8, 16, and 24 hpi. (A) Secreted IL-1β. (B) Intracellular DENV E-protein expression. (C) Infectious virus present in the supernatant, as measured by immunoassay on Vero cells. (D) Relative expression of DENV genome copies in mobilized monocytes, measured by real-time PCR. Values are normalized to 2 hpi samples in the absence of mAb 5G22. For A-D, data are pooled from two independent experiments. (E) Secreted IL-1β by mobilized monocytes at 4 hpi. (F) Secreted IL-1β by fresh, non-mobilized monocytes at 4 hpi. (G) Intracellular DENV E-protein expression at 24 hpi with live DIV crude supernatant or DIV crude supernatant inactivated with formalin or UV exposure, all in the presence of mAb 5G22. (H) Secreted IL-1β at 24 hpi using supernatants from 3G. Tests used: Two-Way ANOVA with Dunnett’s post-test (A–D), One-Way ANOVA with Dunnett’s post-test (G, H), Two-Way ANOVA with Tukey’s post-test (E, F). For A and B, gray (A only) and blue asterisks compare DENV and DENV + 5G22, respectively, to mock within each time point. For C and D, blue asterisks compare DENV + 5G22 to the 2 hpi time point.

As enhancing viral replication had no effect on IL-1β secretion, we determined if the kinetics of IL-1β release differ from the kinetics of viral replication. All previous experiments had assessed IL-1β production at 24 hpi, so we initiated a time course with sample collections at 2, 8, 16, and 24 hpi. Mobilized monocytes inoculated with DIV crude supernatant, with or without mAb 5G22, secreted significantly more IL-1β as early as 8 hpi compared to mock conditions (Fig 3A). However, viral replication only began to elevate significantly at 16 hpi (Fig 3B–3D). Intracellular DENV E-protein expression could not be detected until 16 hpi with DIV crude supernatant in the presence of mAb 5G22 (Fig 3B). To further assess replication of the virus, the amount of infectious virus present in the supernatant (Fig 3C) and intracellular presence of DENV genome copies (Fig 3D) were not significantly increased until 24 hpi with DIV crude supernatant and mAb 5G22. Importantly, none of these measures of DENV replication significantly increased when monocytes were inoculated with DIV crude supernatant in the absence of mAb 5G22. These data confirm that IL-1β secretion induced by DIV crude supernatant precedes replication and is independent of ADE.

As monocytes are reported to secrete IL-1β within 4 hours of inoculation with DENV [19, 20], we next assessed IL-1β secretion at 4 hpi. DIV crude supernatant induced IL-1β secretion by 4 hpi in both mobilized monocytes (Fig 3E) and fresh, non-mobilized monocytes (Fig 3F). These data confirm that IL-1β secretion occurs within 4 hours of inoculation with DIV crude supernatant. Interestingly, though ADE-enhanced IL-1β secretion has been described at 4 hpi [19, 20], ADE was dispensable for IL-1β secretion even at 4 hpi in the current system.

### IL-1β secretion induced by DIV crude supernatant is independent of viral replication

Since IL-1β secretion was elevated before replication could be detected, we sought an alternate way to determine whether infectious DENV was required to induce IL-1β secretion. To do so, we inactivated DIV crude supernatant by incubation with formalin or exposure to shortwave UV irradiation prior to inoculation onto mobilized monocytes. Both formalin and UV inactivation ablated intracellular DENV E-protein expression in mobilized monocytes (Fig 3G). However, each inactivated infectious supernatant induced significant elevation of IL-1β secretion compared to mock conditions (Fig 3H). These data indicate that the replication competency of DENV is dispensable for IL-1β induction by DIV crude supernatant.

### DIV crude supernatant induces *IL1B* and pro-IL-1β expression in primary monocytes

**Fig 4:**
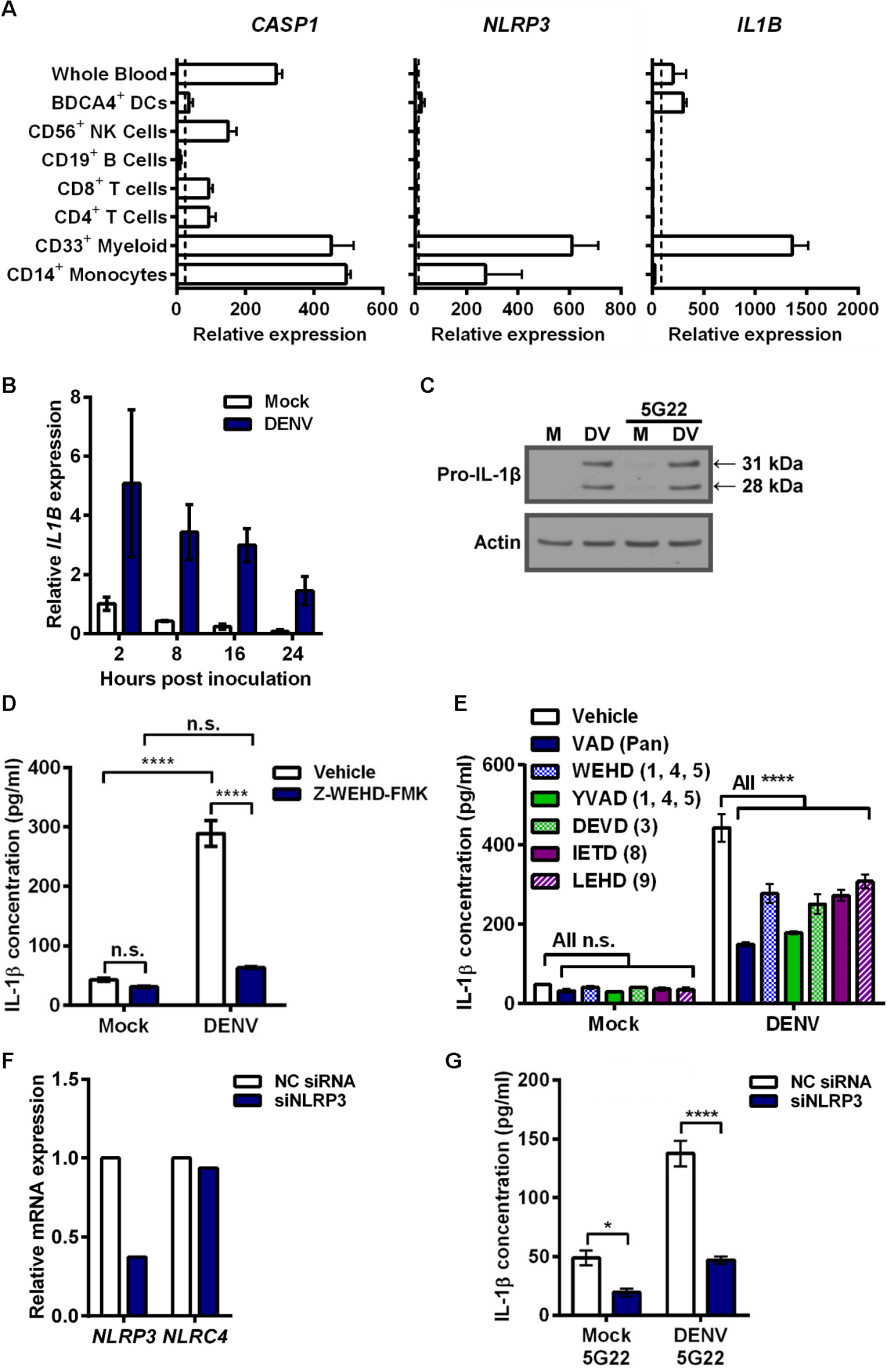
Pro-IL-1β expression induced by DIV crude supernatant requires caspase activity and NLRP3 activation for secretion. (A) Bioinformatic analysis of *CASP1*, *NLRP3*, and *IL1B* gene expression in various human immune cells using the publically-available BioGPS datasets. Data is mean + SD (2 values each) of the measurements for each cell type. Dashed line represents mean of all measured tissues for probe sets listed in *Materials and Methods*. (B) Real-time PCR measurement of relative *IL1B* expression by mobilized monocytes that were lysed at indicated time points after inoculation with DIV crude supernatant. Data (mean ± SD of 2 values per condition) are normalized to 2 hpi mock samples. (C) Immunoblot assessing intracellular pro-IL-1β (31 kDa) in 4 hpi lysates of mobilized monocytes after inoculation with mock supernatant or DIV crude supernatant in the presence or absence of mAb 5G22. (D) Secreted IL-1β by mobilized monocytes at 5 hpi with DIV crude supernatant. Cells were pre-treated with DMSO vehicle or 80 μM of Z-WEHD-FMK caspase-1 inhibitor for 30 minutes prior to inoculation. (E) Secreted IL-1β by mobilized monocytes at 4 hpi with DIV crude supernatant. At time of inoculation, monocytes were treated with DMSO vehicle or 1 μM of various caspase inhibitors: Z-VAD-FMK (pan), Z-WEHD-FMK (1, 4, 5), Z-YVAD-FMK (1, 4, 5), Z-DEVD-FMK (3), Z-IETD-FMK (8), or Z-LEHD-FMK (9). (F) Assessment of *NLRP3* (target) and *NLRC4* (control) expression by real-time PCR, 24 hours after mobilized monocytes were transfected with a control siRNA or an siRNA targeting *NLRP3*. (G) Secreted IL-1β by knockdown and control cells described in 4F at 6 hpi with mock supernatant or DIV crude supernatant in the presence of 1 μg/ml 5G22. Monocytes were inoculated 24 hours after siRNA transfection. Tests used: Two-Way ANOVA with Tukey’s post-test (D), Two-Way ANOVA with Dunnett’s post-test (E), Two-Way ANOVA with Bonferroni’s post-test (G).

Elevated secretion of IL-1β can be caused by increased pro-IL-1β expression, increased inflammasome activation, or both. To assess the mechanism of IL-1β induction by DIV crude supernatant, we first considered known cellular expression of key genes by accessing the online bioinformatics database BioGPS (Fig 4A). CD14^+^ monocytes express high baseline levels of *CASP1* and *NLRP3*, which encode the inflammasome components caspase-1 and NLRP3, respectively. However, basal expression of *IL1B* is low, indicating it likely needs induction. Thus, we measured *IL1B* expression after inoculation of mobilized monocytes with DIV crude supernatant (Fig 4B). *IL1B* expression increased rapidly 2 hours after inoculation with DIV crude supernatant, compared to mock conditions, and gradually reduced over time. Correspondingly, we detected a strong induction of 31-kDa pro-IL-1β expression in the cell lysates of mobilized monocytes collected 4 hours after inoculation with DIV crude supernatant (Fig 4C). As expected from the ELISA results, mAb 5G22 did not enhance the pro-IL-1β expression induced by DIV crude supernatant.

### Caspase activity and NLRP3 are required for IL-1β secretion induced by DIV crude supernatant

As mobilized monocytes processed IL-1β after inoculation with DIV crude supernatant, we next sought to assess inflammasome involvement. We were unable to reproducibly and reliably detect active caspase-1, a notoriously difficult protein to detect in human cells. However, pre-treatment of monocytes with the irreversible caspase-1 inhibitor Z-WEHD-FMK at 80 μM significantly reduced the IL-1β secretion induced by DIV crude supernatant (Fig 4D). As caspase inhibitor peptides can be prone to cross-reactivity, we next expanded our caspase studies to utilize low (1 μM) doses of a broad panel of caspase inhibitors (Fig 4E). Inhibitors targeting all caspases (Z-VAD-FMK), caspases-1, -4, and -5 (Z-WEHD-FMK and Z-YVAD-FMK), caspase-3 (Z-DEVD-FMK), caspase-8 (Z-IETD-FMK), and caspase-9 (Z-LEHD-FMK) all significantly reduced IL-1β secretion induced by DIV crude supernatant. This suggests that either cell death plays an important role in this IL-1β induction or other caspases contribute to inflammasome activation. For example, caspase-8 has been found to be important for inflammasome activation [35, 36].

To test for inflammasome involvement more specifically, we genetically interfered with *NLRP3* expression, as the NLRP3 inflammasome is activated by a wide array of stimuli. We transfected cells for 24 hours with either a negative control siRNA or one targeting *NLRP3* prior to inoculation with DIV crude supernatant (Fig 4F and 4G). At the time of viral inoculation, *NLRP3* expression was reduced approximately 63% by the specific siRNA compared to the negative control (Fig 4F). Expression of *NLRC4*, an alternate NLR family member, was not affected. Knockdown of *NLRP3* expression significantly reduced the secretion of IL-1β by mobilized monocytes after inoculation with DIV crude supernatant (Fig 4G). In sum, these data indicate that caspase activity and NLRP3 activation are required for IL-1β secretion induced by DIV crude supernatant.

### Depletion of antibody-bound virions does not reduce IL-1β secretion induced by DIV crude supernatant

**Fig 5:**
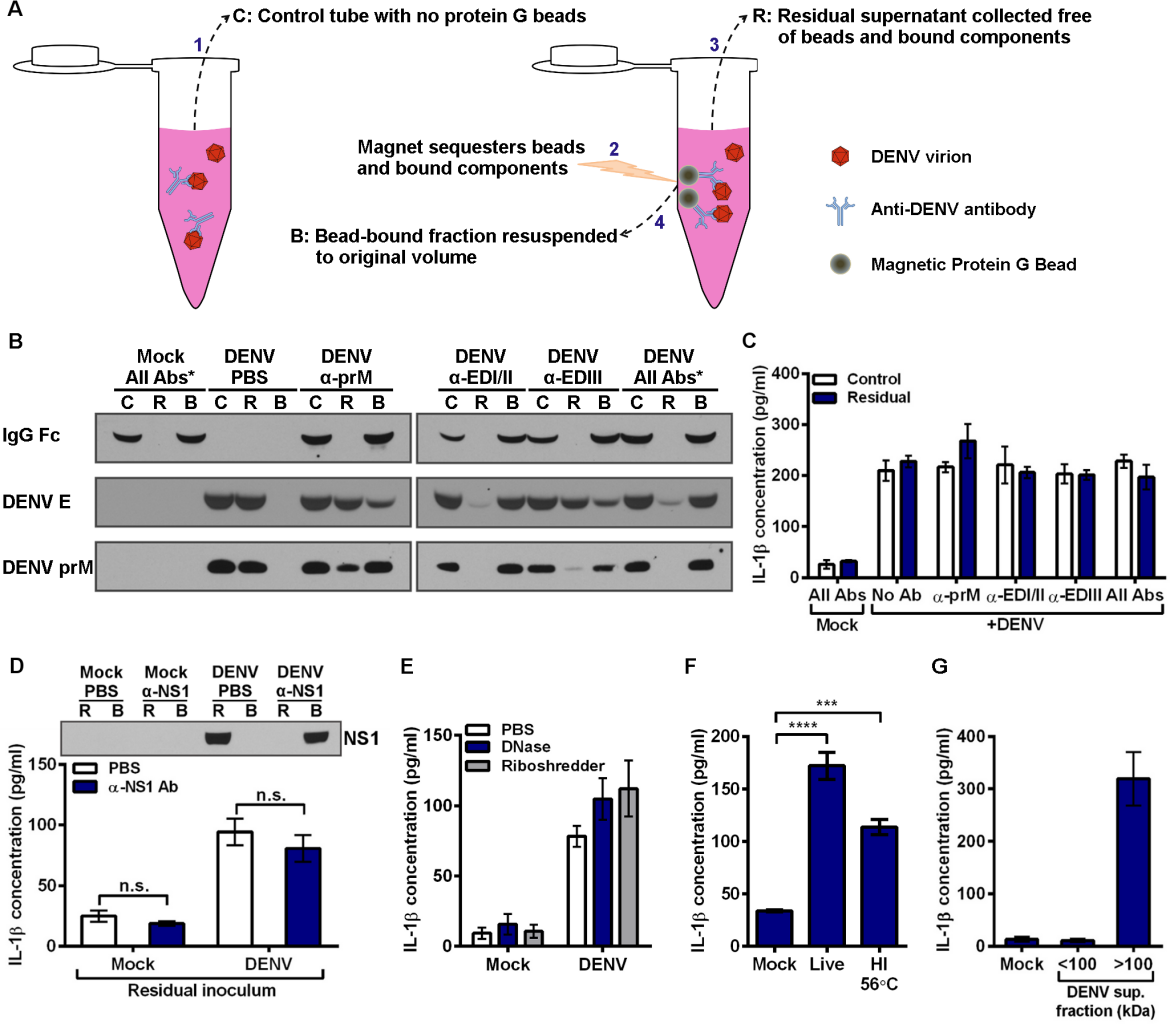
Inflammatory component produced by Vero cells during DENV propagation is responsible for IL-1β induction. (A) Schematic depicting the different depletion and control fractions for depleting antibody-bound virions from DIV crude supernatant by using magnetic protein G beads. (B) Immunoblot for human IgG Fc, and two DENV structural proteins, E protein and prM, to assess efficiency of antibody-mediated DENV depletion from DIV crude supernatant. “*” denotes all 3 antibodies were used in combination at 1 μg/ml each. 3 μg/ml of each antibody was used for individual antibody conditions. (C) Control supernatants (Lane C) and residual supernatants (Lane R) from panel B were inoculated onto mobilized monocytes. Monocyte supernatants were collected at 24 hpi, and secreted IL-1β was measured by ELISA. (D) Mock and DIV crude supernatants were incubated with PBS or anti-DENV NS1 antibody prior to addition of protein G beads and subsequent depletion. Top: Residual supernatants (R) and bead-bound fractions (B) were assessed for efficiency of NS1 depletion by immunoblot. Bottom: Residual supernatants were inoculated onto mobilized monocytes. At 4 hpi, monocyte supernatants were collected and assessed for secreted IL-1β. (E) Mock and DIV crude supernatants were incubated with PBS, DNase, or Riboshredder prior to inoculation onto mobilized monocytes. At 4 hpi, monocyte supernatants were collected and assessed for secreted IL-1β. (F) Mobilized monocytes were inoculated with mock supernatant, live DIV crude supernatant, or DIV crude supernatant that had been incubated for 30 minutes at 56°C to heat inactivate (HI) it. At 24 hpi, monocyte supernatants were collected and assessed for secreted IL-1β. (G) DIV crude supernatant was centrifuged in an Amicon centrifugal filtration unit with a 100-kDa molecular-weight cutoff to generate fractions smaller and larger than 100 kDa. Fractions were brought to equal volumes and inoculated onto mobilized monocytes. At 24 hpi, monocyte supernatants were collected, and IL-1β secretion was measured. Tests used: Two-Way ANOVA with Bonferroni’s post-test (D), One-Way ANOVA with Dunnett’s post-test (F).

We next sought to determine the reason that anti-DENV antibodies can enhance DENV replication without altering the secretion of IL-1β. We considered the possibility that a soluble factor not associated with the virion could induce signaling in the monocytes. To test this, we developed a method of depleting antibody-bound virions from DIV crude supernatant (Fig 5A). Antibodies targeting several DENV epitopes were incubated with DIV crude supernatant individually or in combination. Un-depleted control tubes (fraction C in schematic) received an equal volume of PBS in lieu of beads. Magnetic protein G beads were incubated in depletion tubes. Subsequently, depletion tubes were placed on a magnet, sequestering beads, and all bead-bound components, to the side of the tube. Residual supernatant (fraction R) could then be collected free of bead-bound components. Finally, the bead-bound fraction (fraction B) was resuspended to the original volume for analysis. Depleting monoclonal antibodies had specificity for prM, EDI/II, or EDIII, three main antibody targets found on the surface of the DENV virion, as described previously [37]. Control, residual, and bead-bound fractions were then assessed by immunoblot for efficiency of DENV depletion (Fig 5B). Under mock conditions with all three antibodies, DENV E and prM antigens were not detected, as expected. Human IgG was not detected in the residual fraction depleted of bead-bound components (lane R), while there was strong detection of human IgG Fc in the bead-bound fraction (lane B). This indicates that the protein G beads efficiently removed all human IgG. In a second control containing DIV crude supernatant in the absence of antibodies (DENV + PBS), DENV E protein and prM were only detected in the residual fractions (lane R). This indicates that beads did not non-specifically bind and deplete DENV virions. Next, each antibody was used individually at 3 μg/ml with DIV crude supernatant. This resulted in varying levels of DENV depletion, as assessed by the depletion of DENV E or prM proteins from the residual fractions (lane R). Importantly, the mAb targeting DENV EDI/II removed nearly all detectable virus from the residual supernatant. Similar results were obtained when all three antibodies were added at 1 μg/ml each with DIV crude supernatant. By contrast, anti-prM and anti-EDIII mAbs only partially removed DENV virions when compared to their corresponding control supernatants.

Subsequently, both the un-depleted control supernatants (lane C) and antigen-depleted supernatants (lane R) were inoculated onto mobilized monocytes at an approximate pre-depletion MOI of 50 (Fig 5C). No depletion condition altered IL-1β secretion compared to its respective un-depleted control supernatant. These data suggest that DENV virions are not responsible for the IL-1β secretion induced by DIV crude supernatant.

### Several potential factors eliminated as IL-1β-inducing component of DIV crude supernatant

To identify the component in DIV crude supernatant that could induce IL-1β secretion, we investigated several possibilities. First, though most DENV nonstructural proteins are neither present in an infectious virion nor secreted from infected cells, nonstructural protein 1 (NS1) is secreted from infected cells as a soluble hexamer [38]. To determine if soluble NS1 present in DENV supernatant is responsible for the induction of IL-1β secretion, we depleted NS1 from viral supernatant using antibody-mediated depletion with protein G beads, which resulted in the near-complete removal of NS1 from DIV crude supernatant (Fig 5D). However, inoculating mobilized monocytes with NS1-depleted DIV crude supernatant had no impact on the secretion of IL-1β.

We next verified that DIV crude supernatants were not contaminated with LPS, a potent IL-1β agonist. Two independent DIV crude supernatant samples were sent to the UNC Tissue Culture Facility for LPS screening by a limulus amebocyte lysate assay. Both samples were reported to contain less than or equal to 0.10 Endotoxin Units/ml, thus excluding LPS contamination as a factor.

To investigate the possibility that DENV-infected Vero cells release inflammatory RNA or DNA, we treated DIV crude supernatant with DNase or RNase prior to inoculation onto mobilized monocytes (Fig 5E). Neither nuclease prevented IL-1β secretion induced by DIV crude supernatant. Complement has been associated with the response to DENV infection [39]. To assess if complement or another heat-sensitive component had a role, DIV crude supernatant was heated for 30 minutes at 56°C prior to inoculation onto mobilized monocytes (Fig 5F). Heat-inactivated DIV crude supernatant still induced elevated IL-1β secretion over mock conditions.

To test if cytokines secreted by infected Vero cells, which originated from African green monkey kidney, could induce IL-1β secretion by primary human monocytes, we filtrated the DIV crude supernatant. Ultrafiltration of human plasma using a pore with a 60-kDa molecular-weight cutoff (MWCO) has been shown to effectively remove IL-1β, IL-6, TNFα, IL-10, and IL-8, as these cytokines are smaller and able to pass through the pore [40]. Thus, we chose to employ centrifugal filtration with a MWCO of 100 kDa to allow flow-through of cytokines but not DENV or other larger components. The flow-through fraction, containing everything less than 100 kDa (approximately), and the concentrated retentate (components above 100 kDa) were collected and brought to equal volumes. Subsequently, equal volumes of both fractions and a mock condition were inoculated onto mobilized monocytes (Fig 5G). Only the fraction containing components larger than 100 kDa induced the secretion of IL-1β by primary monocytes. This excluded many key cytokines present in Vero-derived supernatant as a contributing factor to IL-1β induction.

### Antibody promotes DENV-induced IL-1β secretion by primary monocytes infected with purified virus

**Fig 6:**
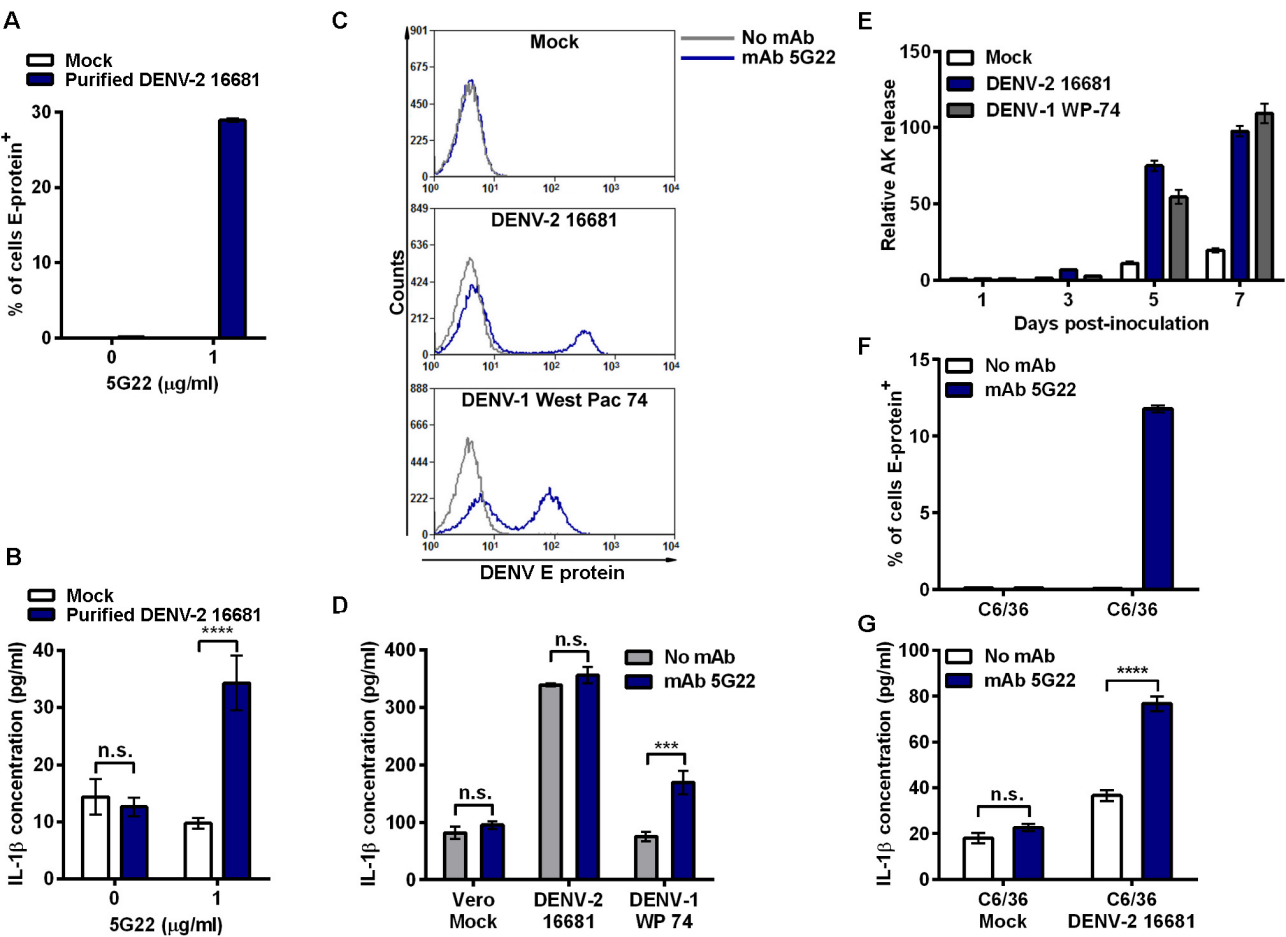
ADE-induced IL-1β secretion varies by DENV purity, strain, and cell-type used for propagation. (A) Mobilized monocytes were inoculated with mock conditions or purified DENV-2 16681 derived from Vero cells with or without the addition of mAb 5G22. Intracellular DENV E-protein expression was measured at 24 hpi. (B) Secreted IL-1β detected in 24-hpi monocyte supernatants from 6A. (C) Monocytes were inoculated with DIV crude supernatants harvested from Vero cells infected with DENV-2 16681 or DENV-1 West Pac 74 with or without mAb 5G22. Cells were washed at 1 hpi, and intracellular DENV E-protein expression was measured at 24 hpi. (D) IL-1β in 24-hpi supernatants from 6C was measured by ELISA. (E) Vero cells were inoculated with MOI 0.5 of DENV-2 16681 or DENV-1 West Pac 74. At 1 hpi, inoculum was aspirated and fresh medium was added. Supernatant samples were collected at days 1, 3, 5, and 7 post-inoculation and assessed for adenylate kinase release by ToxiLight assay. Values are normalized to the 1 dpi mock condition. (F) Mobilized monocytes were inoculated with control supernatant from uninfected C6/36 cells or supernatant derived from C6/36 cells infected with DENV-2 16681, with or without 1 μg/ml mAb 5G22. Cells were washed at 1 hpi, and intracellular DENV E-protein expression was analyzed at 24 hpi. (G) Secreted IL-1β detected in 24-hpi monocyte supernatants from 6E. Tests used: Two-Way ANOVA with Bonferroni’s post-test (B, D, G).

Though the identity of the inflammatory component present in DIV crude supernatant remained unclear, we wanted to assess if purification of DENV virions away from other supernatant components would alter the secretion of IL-1β. To purify the virions, DIV crude supernatant was concentrated and subjected to ultracentrifugation through a 10%–40% continuous sucrose gradient [41]. Subsequently, mobilized monocytes were inoculated with purified DENV-2 16681 alone or with mAb 5G22. Similar to the finding with DIV crude supernatant, intracellular DENV E-protein was only detected in monocytes at 24 hpi in the presence of mAb 5G22 (Fig 6A). However, unlike that induced by DIV crude supernatant, purified DENV-2 16681 derived from Vero cells induced significant elevation of IL-1β secretion only in the presence of mAb 5G22 (Fig 6B). Interestingly, the level of IL-1β induced by purified DENV is lower than that induced by DIV crude supernatant shown in earlier experiments, consistent with the presence of an additional inflammatory component in the crude supernatant.

### IL-1β induction varies by DENV strain and cell-type used to propagate virus

We next assessed whether a different DENV strain propagated in Vero cells would similarly induce ADE-independent IL-1β secretion. In addition to DENV-2 16681, monocytes were also inoculated with DIV crude supernatant harvested from Vero cells infected with DENV-1 West Pac 74, in the presence or absence mAb 5G22. At 24 hpi, both DENV strains induced intracellular E-protein expression only when inoculated in the presence of mAb 5G22 (Fig 6C). Interestingly, the induction of IL-1β varied dramatically by strain (Fig 6D). As before, DENV-2 16681 DIV crude supernatant induced ADE-independent IL-1β secretion. However, DENV-1 West Pac 74 DIV crude supernatant induced IL-1β only under ADE conditions. These data indicate that IL-1β induced by DIV crude supernatant can vary by DENV strain.

Though grown in Vero cells under the same conditions, we found DENV-1 West Pac 74 to grow to titers approximately three-fold higher than DENV-2 16681. Thus, inoculating monocytes with equal MOIs of the two strains requires a lower volume of DIV crude supernatant for DENV-1 West Pac 74. To see if these DENV strains induce cell death in Vero cells, we inoculated Vero cells with equal MOIs of both DENV strains and monitored adenylate kinase (AK) release over time compared to mock inoculation (Fig 6E). Both DENV strains induced a similar degree of cell death in Vero cells compared to the mock condition. Thus, increased cell death alone cannot account for the masking phenotype seen when inoculating monocytes with DENV-2 16681. However, the lower volume of DIV crude supernatant required to inoculate monocytes with DENV-1 West Pac 74 may reveal ADE-induced IL-1β by exposing monocytes to a reduced amount of the inflammatory components.

Other studies describing ADE-induced IL-1β secretion in primary monocytes utilized crude supernatant harvested from DENV-infected C6/36 mosquito cells [19, 20]. We next verified that crude supernatant harvested from C6/36 cells infected with DENV-2 16681 induced ADE-dependent IL-1β secretion. Crude supernatant harvested from C6/36 cells infected with DENV-2 16681 was inoculated onto mobilized monocytes in the presence or absence of mAb 5G22. Similar to Vero-derived DENV-2 16681, mAb 5G22 enhanced the intracellular expression of DENV E protein in mobilized monocytes 24 hours after inoculation with crude infectious supernatant from C6/36 cells (Fig 6F). As expected based on our previous study [20], ADE with mAb 5G22 significantly enhanced IL-1β secretion when mobilized monocytes were inoculated with crude supernatant from C6/36 cells infected with DENV-2 16681 (Fig 6G). Importantly, the presence of mAb 5G22 did not impact IL-1β secretion induced by crude supernatant from uninfected C6/36 cells. These data suggest that, unlike Vero cells, C6/36 mosquito cells do not produce a potent inflammatory component during the propagation of DENV-2 16681 in culture. Instead, DENV-2 16681 propagated in mosquito cells displayed ADE of both infection and IL-1β secretion.

## Discussion

This work shows that while all DENV preparations display an identical requirement for ADE to enhance viral replication, the source and purity of DENV preparations greatly impact the induction of IL-1β secretion by primary human monocytes. DENV-2 16681 propagation in Vero cells produced not only high titers of infectious virus, but also inflammatory moieties that induced IL-1β secretion. Initial experiments utilized debris-cleared supernatant derived from DENV-infected Vero cells, a common method of viral preparation to analyze the immune response to DENV [42–46]. This Vero-derived supernatant displayed a strong IL-1β-inducing activity that was unaltered by ADE, despite concurrent enhancement of infection by antibody. Crude supernatant harvested from a second, independent line of Vero cells infected with DENV-2 16681 similarly induced ADE-independent IL-1β secretion. Induction of other inflammatory cytokines was also independent of ADE. This indicates that an unknown factor produced by DENV-infected Vero cells may mask the DENV-induced production of numerous cytokines.

Once DENV-2 16681 virions propagated in Vero cells were purified and separated from other supernatant components, a different phenotype emerged. Purified DENV-2 16681 from Vero cells induced significantly more IL-1β secretion by monocytes in an ADE-dependent fashion. ADE similarly enhanced IL-1β secretion when inoculating monocytes with crude supernatant from C6/36 mosquito cells infected with DENV-2 16681. This indicates that the inflammatory moiety in Vero-derived supernatant that caused IL-1β secretion is not produced by mosquito cells. As inoculation with crude supernatant from DENV-infected Vero cells is commonly used in the field, precaution should be exercised in using such a preparation to study the DENV-induced inflammatory response in immune cells. However, this phenotype was not universal amongst DENV strains tested. Crude supernatants harvested from Vero cells infected with DENV-1 West Pac 74 induced ADE-dependent IL-1β. This is possibly because this virus grows to high titers and thus less virus-containing supernatant was added to cells.

Inoculation of primary mobilized monocytes with crude supernatant from Vero cells infected with DENV-2 16681 rapidly induced the expression of *IL1B* and pro-IL-1β. Thus, a key step leading to elevated IL-1β secretion after inoculation with this supernatant appears to be the enhancement of pro-IL-1β expression. Although bioinformatic analysis with BioGPS shows that *CASP1* and *NLRP3* transcripts are constitutively expressed by human monocytes, inhibition of caspase-1 with a pharmacologic inhibitor or NLRP3 with RNA interference reduced the secretion of IL-1β, indicating involvement of the NLRP3 inflammasome.

Although much effort was devoted to identifying the IL-1β-inducing moiety or moieties in DIV crude supernatant, the nature of the inducer(s) remained elusive. Could it be a cell-culture artifact that needs to be eliminated from conventional crude viral preparations? Or is this a physiologically-relevant component (either viral- or host-derived) that merits further studying? Our data suggests that the factor is larger in size than many key cytokines, is heat stable at 56°C, and is not RNA, DNA, or LPS contamination. Further, immunodepletion studies confirm that NS1 is not responsible for the IL-1β induction. Of note, one study has shown that Vero cells infected with DENV undergo more apoptosis than infected C6/36 mosquito cells [47]. It is possible that by-products of increased cell death may alter the inflammatory phenotype of crude supernatant from DENV-infected Vero cells.

The IL-1β-inducing factor present in crude supernatant from Vero cells infected with DENV-2 16681 likely masked the response induced by DENV and anti-DENV antibody in the current system. This is an important point to consider, as it can generate misleading results. Consistent with our results using DENV-1 West Pac 74, successful achievement of ADE-induced cytokines by other groups using crude supernatant derived from DENV-infected Vero cells indicates that this inflammatory phenotype is not universally produced by all Vero cells or in all systems [43, 45]. However, a masking component should be considered when disjointed results between infection and cytokine production are found, particularly under ADE conditions. We have previously shown [20] and confirmed here that crude supernatant from DENV-infected C6/36 cells does not exhibit these confounding issues and represents a more straightforward system to study the ADE-induced inflammatory response in human immune cells. Finally, though the use of purified virus effectively removed the inflammatory moiety from DIV crude supernatant, this approach is much more labor-intensive and unlikely to be used routinely.

## Materials and Methods

### Ethics Statement

Non-mobilized PBMCs were isolated from fresh blood drawn from a de-identified healthy donor under Study #13–2115 approved by the University of North Carolina at Chapel Hill Institutional Review Board and Office of Human Research Ethics, with written informed consent provided. Mobilized peripheral blood mononuclear cells (PBMCs) were isolated from the blood of leukapheresed patients enrolled in Study #05–2860 approved by University of North Carolina at Chapel Hill Institutional Review Board and Office of Human Research Ethics after providing written informed consent. Samples were anonymized and provided as de-identified samples prior to use in the described studies. The University of North Carolina at Chapel Hill Office of Human Research Ethics determined that the use of the de-identified samples does not constitute human subjects research as defined under federal regulations [45 CFR 46.102 (d or f) and 21 CFR 56.102(c)(e)(l)] and does not require further Institutional Review Board approval.

### PBMC isolation and cell culture

Due to the large number of cells required for this study, we mostly employed cryopreserved, primary mobilized PBMCs from two separate patients injected with G-CSF, which greatly increases the number of circulating leukocytes, for further studies [48]. This established a large stock of monocytes isolated in one day that was capable of supplying months of experiments, reducing the inherent variability of human studies. We then verified key phenotypes with PBMCs from freshly-isolated, non-mobilized blood.

PBMCs were isolated from the blood using a 1.073 g/ml Ficoll-Hypaque Premium gradient (GE Healthcare) to enhance monocyte isolation. The manufacturer’s suggested protocol was followed for buffy coat isolation. Negative isolation was done using the Dynabeads Untouched Human Monocytes kit (Invitrogen) by following manufacturer’s protocol. Antibodies against CD3, CD7, CD16 (specific for CD16a and CD16b), CD19, CD56, CDw123, and CD235a depleted cells expressing these markers. Mixed PBMCs and purified monocytes were cryopreserved in 90% heat-inactivated FBS (FBS-HI) with 10% DMSO. On experimental days, cells were thawed, washed twice, and placed at 37°C for 2 hours in PBMC cell culture media consisting of RPMI with 10% FBS-HI, 1% L-glutamine, 1% NEAA, 1% penicillin/streptomycin, and 30 units/ml DNase to prevent cell clumping from the release of DNA by dying granulocytes. Cells were counted and resuspended in PBMC medium without DNase for use in experiments.

The majority of experiments used DENV propagated in the Vero 76 cell line (ATCC CRL-1587). Confirmation of the inflammatory phenotype with an independent line of Vero cells was done using DENV propagated in the original Vero cell line, acquired from the UNC Lineberger Comprehensive Cancer Center Tissue Culture Facility (ATCC CCL-81). The *Aedes albopictus* C6/36 cell line was acquired from ATCC (CRL-1660).

### DENV-specific antibodies

Crude supernatant of mAb D14G2 (4G2), a pan-flavivirus E-protein specific mouse IgG2a mAb, for use in IHC viral titrations was kindly provided by Dr. Mariano Garcia-Blanco of Duke University. Purified mAb 4G2 was generated by the UNC Antibody Core Facility. The isolation and purification of human mAbs 5G22 (α-prM used for enhancement and depletion), 2D22 (α-DENV-2 used for enhancement and neutralization), 6B22 (α-EDI/II used for depletion), 1C17 (α-EDIII used for enhancement and depletion), 1F4 (α-DENV-1 used for isotype-matched control) and 2H21 (α-prM used for immunoblots) were described in detail previously [32, 33].

### Virus stock growth

All experiments used DENV-2 strain 16681, kindly provided by Dr. Robert Tesh of UTMB-Galveston, unless otherwise noted. Vero cells were cultured at 37°C with 5% CO_2_ in MEM + 6% FBS-HI, 1% penicillin/streptomycin, and 20 mM HEPES buffer. To generate large stocks for experiments, near-confluent Vero cell culture monolayers were inoculated with DENV-2 16681 or DENV-1 West Pac 74 at an MOI of 0.5 in low-volume, low-serum conditions, placed in the incubator, and rocked every 15 minutes. At 2 hpi, culture medium was added. For viral stocks grown for purification, 1% FBS-HI was used so as not to clog the centrifugal filters during the concentration step. At days 3, 7, and 10, medium was collected from the flasks and centrifuged at 4,000 RPM for 10 minutes to clarify the supernatants. Infectious crude supernatants were then aliquoted into tubes for freezing at -70°C. Fresh medium replaced the collected supernatants. C6/36 cells were cultured at 29.5°C with 5% CO_2_ in MEM + 10% FBS-HI, 1% NEAA, 1% penicillin/streptomycin, and 20 mM HEPES. Propagation of DENV in C6/36 cells was done as with Vero cells except for listed differences in medium composition.

We modified a previously described protocol to quantitate the infectious titer of viral stocks [49]. Briefly, near-confluent Vero cell monolayers in flat-bottom, 96-well plates were inoculated with 50 μl of sequential 10-fold dilutions of DENV stocks. At 2 hpi, an overlay of 150 μl of 1.6% carboxymethylcellulose (diluted 1:1 in 2X MEM and supplemented with 1% FBS-HI, 10 mM HEPES and 1X antibiotics) was added to limit spread of virus. At 72 hpi, cells were fixed using a 1:1 mixture of acetone and methanol. Fixed cells were blocked with 2% normal horse serum in PBS and subsequently stained for 1 hour using mAb 4G2 as the primary antibody (1:500 dilution of crude supernatant into blocking solution) and 1:1000 goat anti-mouse IgG-HRP (KPL) as the secondary antibody. Viral foci were visualized using Vector VIP Peroxidase Substrate kit (Vector Laboratories, Inc.) and counted under a dissecting microscope, with titers calculated as Vero focus forming units per ml (ffu/ml).

### Inoculation of PBMCs or monocytes

One hour pre-inoculation, dilutions of enhancing antibodies or control medium were plated into 96-well, round-bottom, non-tissue-culture treated plates. Crude infectious supernatants or purified DENV preparations were diluted to the appropriate multiplicity of infection (MOI) and mixed with antibodies or control medium. DENV and antibody mixtures were incubated for 1 hour at 37°C with 5% CO_2_ to allow immune complex formation. For mock infection wells, spent culture medium from uninfected Vero cell or C6/36 cultures was used instead of infectious supernatant. MEM with 1% FBS-HI, 1% penicillin/streptomycin, and 20 mM HEPES was used as the mock condition for purified virus. After the 1 hour incubation for complex formation, mixed PBMCs or purified primary monocytes were added. At 1–2 hours post-inoculation (hpi), cells were washed at least 2 times with PBS and resuspended in fresh culture medium. At collection, cells were resuspended and pelleted by centrifugation. After two washes with 1X PBS (four for genome copy studies), cells were fixed with Cytofix/Cytoperm (BD Biosciences) for 20 minutes at 4°C for flow cytometry, lysed with RLT Lysis Buffer (Qiagen) for RNA analysis, or lysed with 1X RIPA buffer (Boston BioProducts) with protease inhibitors (Roche) for western blot analysis. Monocyte supernatants were centrifuged to clear cells and debris, recollected, and stored at 4°C until analysis by ELISA. Unless otherwise noted, all monocyte inoculations were done with an MOI of 50.

### Flow cytometry

Purified mAb 4G2 was conjugated to Alexa Fluor 647 using the Alexa 647 Protein Labeling Kit (Life Technologies) according to manufacturer’s instructions. After labeling, the labeled antibody was titrated by staining known DENV-positive monocytes to identify the best dilution for detection of DENV E protein without high levels of non-specific background in mock-inoculated cells.

For detection of DENV E protein, cells were washed twice with Perm/Wash buffer (BD Biosciences) after fixation and re-suspended in 25 μl of Human Fc Receptor Binding Inhibitor (eBiosciences) diluted 1:5 in Perm/Wash Buffer for 15 minutes at 4°C. Cells were then incubated with 25 μl of mAb 4G2 conjugated to Alexa Fluor 647 diluted 1:250 in Perm/Wash buffer for 30 minutes at 4°C. Cells were then washed twice with Perm/Wash buffer and finally re-suspended in 200 μl of Perm/Wash buffer for analysis on a Cyan ADP flow cytometer (Dako). Cells were initially gated on FSC-area vs. SSC-area, with single cells positively selected for by gating cells on the diagonal of FSC-height vs. FSC-area. Cells positive for DENV E-protein were detected on the APC channel, with positive gates set based on the mock-inoculated controls.

For surface detection of DENV E protein, cells at 24 hpi were washed with PBS and then resuspended in 25 μl of Human Fc Receptor Binding Inhibitor diluted 1:5 in FACS buffer (2% FBS in PBS) and incubated for 15 minutes at 4°C. Cells were then incubated with 25 μl of mAb 4G2 conjugated to Alexa Fluor 647 diluted 1:250 in FACS buffer for 30 minutes at 4°C. Cells were then washed twice with FACS buffer and resuspended in 50 μl of fresh PBS. Cells were then resuspended with 50 μl of 2% formalin diluted into PBS and allowed to incubate at room temperature for 20 minutes protected from light. Cells were then washed twice with FACS buffer and resuspended with 200 μl fresh FACS buffer. Stained cells were stored at 4°C protected from light until analysis by flow cytometry.

For staining of surface CD14 expression in conjunction with intracellular DENV E-protein expression, cells were washed twice with PBS and incubated at 4°C with Human Fc Receptor Binding Inhibitor in eFluor NC Flow Cytometry Staining Buffer (eBiosciences) supplemented with 2% FBS-HI for 15 minutes at 1 test per well. Cells were then stained with anti-human CD14 eFluor 605NC mouse mAb (eBiosciences) at 1 test size per well in 25 μl 1X staining buffer and incubated for 30 minutes in the dark at 4°C. Cells were washed twice with staining buffer, fixed, permeabilized, and stained with mAb 4G2 conjugated to Alexa Fluor 647, as described above. An unstained control and single-stained controls were set up for gating analysis. For compensation, antibodies were added to anti-mouse BD CompBeads at same dilution as sample staining. Cells and beads were run on a LSRII flow cytometer (Becton Dickinson), using the APC channel for the anti-DENV antibody and QDot605 for the anti-CD14 antibody. Compensation calculations and analysis were done using FlowJo.

### Real-time PCR

After lysis with RLT buffer, cell lysates were passed through QIAshredder columns (QIAGEN) to homogenize lysates. Homogenized lysates were then added to RNA isolation columns from RNeasy Mini kits (QIAGEN), and all steps were followed as detailed in the manufacturer’s protocol. RNA was eluted in a volume of 30 μl RNase/DNase free water. To generate cDNA, 1 μl Random Primers (3 μg/μl; Invitrogen) and 1 μl dNTP Mix (10 mM; Invitrogen) was added to 10 μl of eluted RNA and heated at 65°C for 5 minutes in a PTC-225 Peltier Thermal Cycler (MJ Research). Tubes were chilled on ice and 4 μl Invitrogen 5X First Strand Buffer, 2 μl Invitrogen 0.1M DTT, and 1 μl Promega RNasin RNase Inhibitor (40 units/μl) were added to each tube and incubated at 37°C for 2 minutes. Then 1 μl Invitrogen M-MLV Reverse Transcriptase (200 units/μl) was added per tube, and tubes were incubated at 25°C for 10 minutes and 37°C for 50 minutes, with an inactivation step of 70°C for 15 minutes.

For real-time PCR analysis, 9 μl cDNA was added to 1 μl 20X TaqMan Gene Expression Assay mix (Applied Biosystems [ABI]) and 10 μl of ABI 2X TaqMan Universal PCR Master Mix in a 384-well plate. Samples were pipetted in triplicate. Plates were run on an ABI 7900 HT Fast Real-Time PCR machine using the following parameters: 50°C for 2 minutes, 95°C for 10 minutes, and 40 repeats of 95°C for 15 seconds followed by 60°C for 1 minute. Fold change values were calculated using the ΔΔCt method, normalized to a control value set at 1. Where applicable, all biological replicates (including their corresponding pipetting triplicates) were combined and outliers greater than 2 standard deviations away from the mean were excluded. For all samples, 18s rRNA was used as the housekeeping-gene control.

The following ABI TaqMan Gene Expression Assays were used for real-time PCR analysis of gene expression: assay Hs01555410_m1 was used for *IL1B*, Hs00918082_m1 was used for *NLRP3*, Hs00368367_m1 was used for *NLRC4*, and Hs03928985_g1 was used for *RN18S1*. For DENV genome copies, a custom TaqMan Gene Expression Assay was designed using nucleotides 10635–10708, a portion of the 3′ UTR of DENV conserved between all 4 serotypes, identified and described by Gurukumar, *et al*. [50].

### Caspase inhibition

For inhibition of caspase-1 only, Z-WEHD-FMK Caspase-1 Inhibitor (R&D Systems) was diluted in sterile DMSO and cells were pre-treated with 80 μM Z-WEHD-FMK or DMSO vehicle 30 minutes prior to inoculation with DENV. Dilutions were made into cell culture medium.

For the caspase inhibition panel, Z-VAD-FMK (pan caspase inhibitor), Z-WEHD-FMK and Z-YVAD-FMK (caspases-1, -4, and -5 inhibitors), Z-DEVD-FMK (caspase-3 inhibitor), Z-IETD-FMK (caspase-8 inhibitor), and Z-LEHD-FMK (caspase-9 inhibitor) (all from R&D Systems) were diluted in sterile DMSO and given to the cells at a final concentration of 1 μM each at the time of inoculation with DIV crude supernatant.

### Bioinformatic analysis

The publicly available BioGPS expression database was used for all bioinformatic analysis [51]. The U133A, gcrma dataset was used for analysis of the following genes: *CASP1* (probeset 206011_at), *NLRP3* (probeset 207075_at), and *IL1B* (probeset 205067_at) [52].

### Gene knockdown

Knockdown of *NLRP3* was achieved using QIAGEN’s HiPerFect Transfection Reagent and FlexiTube GeneSolution GS114548 for NLRP3. QIAGEN’s AllStars Negative Control siRNA served as a negative control for *NLRP3* knockdown. Briefly, based upon manufacturer’s instructions, concentrations of both transfection reagent and siRNAs were optimized in a pilot experiment. Knockdown was analyzed by decrease of *NLRP3* expression as analyzed by real-time PCR compared to control. For the experimental knockdown, 4 x 10^6^ cells per condition were plated in 7 ml of growth medium in a 150 mm dish. 8 μl of each 10 mM stock of NLRP3 siRNAs was added to 1 total ml of serum-free RPMI (16 μl of 20 mM negative control siRNA). Then, 20 μl of HiPerFect transfection reagent was added to each tube, vortexed, and allowed to incubate at room temperature. After 10 minutes, complexes were dripped onto the cells, plates were swirled, and allowed to incubate for 24 hours. At 24 hours post-transfection, cells were collected, counted, and immediately used for infection assays.

### SDS-PAGE and immunoblots

For protein studies to be analyzed by immunoblot, primary monocytes were lysed in the 96-well plates using RIPA Buffer (Boston BioProducts) containing 1X Complete Protease Inhibitor (Roche). Plates were rocked at 4°C for 20 minutes. Lysates were collected and centrifuged at 13,000 RPM for 10 minutes to clear debris. Lysates were recollected into fresh tubes. 4X NuPAGE LDS Sample Buffer (Life Technologies), containing 20 mg/ml DTT reducing reagent (Roche), was added to a final concentration of 1X. Samples were mixed and heated at 97°C for 5 minutes.

For viral protein analysis by immunoblot, NuPAGE LDS Sample Buffer without DTT was added directly to DENV-containing supernatant to a final concentration of 1X. Samples were vortexed and heated at 97°C for 10 minutes. DTT was added to samples at above concentrations for detection of DENV NS1 by immunoblot.

Samples were loaded in NuPAGE 4–12% Bis-Tris pre-cast gels (Life Technologies) and separated by SDS-PAGE using 1X NuPAGE MES SDS running buffer (Life Technologies). Gels were transferred onto 0.2 μm nitrocellulose membranes (Bio-Rad) under wet transfer conditions using 1X Transfer Blotting Buffer (Boston BioProducts) with 30% methanol for 45 minutes at a constant 100V. Membranes were blocked in a 10% milk solution in TBS-T for 1 hour at room temperature. Primary antibodies were incubated overnight at 4°C with rotation at the following dilutions in blocking buffer: 1:1,000 rabbit polyclonal antibody anti-IL-1β (Santa Cruz sc-7884), 1 μg/ml purified mouse mAb 4G2 to detect DENV E protein, 1 μg/ml human mAb 2H21 to detect DENV prM, 1:1000 goat anti-human Fc conjugated to HRP (Bethyl Laboratories) to detect human IgG Fc, or 1:1000 rabbit anti-DENV NS1 polyclonal antibody (Genetex GTX103346). After 4 washes with TBS-T, the membranes were incubated with the following secondary antibodies diluted into blocking buffer for 2 hours at room temperature: 1:5000 goat anti-rabbit HRP-conjugate (Santa Cruz) for IL-1β and NS1, 1:5000 goat anti-mouse HRP-conjugate (Santa Cruz) for DENV E protein, or 1:5000 goat anti-human HRP conjugate (Bethyl) for DENV prM. Human IgG in depletion studies was detected directly after primary antibody incubation because of HRP conjugate. Membranes were then washed 4–5 times with TBS-T and subsequently developed after 5 minute incubations with Thermo Scientific SuperSignal West Pico Chemiluminescent Substrate.

### Depletion of antibody-bound virions and NS1

Mock and DIV crude supernatants were incubated with indicated concentrations of antibodies (or equal volume of PBS control) for 1 hour on ice. Subsequently, either 50 μl of pre-washed Dynabeads Protein G beads (Life Technologies) or equal volume of PBS were added to samples, and placed on a rotator at 4°C for 1 hour. Tubes were then placed onto a DynaMag-2 magnet for 1 minute, and bead-free supernatants were collected and transferred to a new tube. Placement on the magnet with collection of the supernatant was repeated two more times to ensure complete removal of beads. The bead-free supernatants beads were considered the residual fractions, while the resuspended beads (for protein analysis only) were considered the bead-bound fractions. Tubes that had PBS added in lieu of beads were considered the control fractions, as equal volumes were maintained but no antigens were depleted. Depletion of NS1 was done identically by using mouse anti-NS1 monoclonal antibody clone DN1 (Abcam #ab41490) to immunoprecipitate NS1 with protein G beads. Monoclonal antibody DN1 was washed through an Amicon filter to remove preservatives, resuspended in PBS at a 6-fold increase in concentration, and then syringe filtered to purify. This was then used at a final dilution of 1:2 for successful depletion of NS1.

### DNase and RNase treatment of viral supernatant

Mock and DIV crude supernatants were incubated with 30 units/ml of amplification grade DNase I (Life Technologies), 6 units/ml RiboShredder RNase Blend (Epicentre), or an equal volume of PBS. Samples were incubated for 20 minutes at room temperature for DNase activity, 20 minutes at 37°C for RNase activity, and finally 12 minutes at 65°C followed by 2 minutes at 97°C to ensure inactivation of the enzymes prior to inoculation onto cells.

### Virus purification

To purify virus, a previously described protocol was used [41]. Briefly, crude supernatants from DENV-infected Vero cells were concentrated by centrifugation at 1500 x g for 20–25 minutes in 15 ml Millipore Amicon Centrifugal Filter Units with a 100-kDa cutoff, allowing components < 100 kDa to pass through but not virus. The final concentrated volume was approximately 2 ml, which was gently layered on top of an 8-ml 10% ‒ 40% continuous sucrose gradient in an ultracentrifuge tube. The gradient was centrifuged in a Beckman Coulter Optima L-90K Ultracentrifuge using rotor SW40TI for 2.5 hours at 35,000 RPM and 4°C with no brake. Sequential 0.5 ml fractions were collected from the bottom of the ultracentrifuge tube into microfuge tubes. Fractions 4–8 were pooled and washed to remove sucrose using another 15 ml Amicon tube. After 2 washes, the virus was resuspended in MEM with 1% FBS-HI, 20 mM HEPES, and 1% penicillin/streptomycin. The pooled fractions were then titered as described earlier.

### Virus inactivation

DIV crude supernatant was inactivated using formalin or shortwave UV exposure. For formalin inactivation, 0.25% formalin (final concentration) was added to an aliquot of virus stock and allowed to incubate at room temperature for 2.5 hours. For shortwave UV inactivation, 100 μl/well of virus stock was added to each well of a 24-well plate, placed on ice, and exposed to shortwave UVB (254 nm) irradiation for 2 min at a distance of approximately 5 cm. After inactivation, the inactivated supernatants, and an equal volume of live virus, were added into separate 15 ml Amicon tubes and washed twice using fresh medium to remove formalin (or as control for sample loss for supernatants not treated with formalin). After washing, the supernatants were resuspended in fresh culture medium to equal volumes and inoculated onto monocytes.

### Cytokine detection

Detection of human IL-1β in the supernatant was done using BD Biosciences’ BD OptEIA Human IL-1beta ELISA Set II. The kit was followed as per manufacturer’s instructions, and the absorbance at 450 nm was read on a PerkinElmer EnSpire Multimode Reader 2300. Wavelength correction at 570 nm was used.

Detection of human IL-1β, IL-12, TNF, MIP-1α, and MIP-1β for Fig 2B–2F was done by assaying 50 μl of 24 hpi supernatants in the Invitrogen Human Cytokine 25-Plex Luminex Bead Panel according to manufacturer’s instructions in the Regional Biocontainment Laboratory at Duke Immunology Unit (Duke University, Durham, NC).

### Adenylate kinase release

Adenylate kinase release into culture supernatants was assessed by ToxiLight (Lonza). Supernatants were stored at -70°C until the assay was run. Then, 20 μl of culture supernatant was mixed with 100 μl of adenylate kinase detection reagent. After 5 minutes, luminescence was measured by plate reader. Cell-free media controls collected at each time point were subtracted from each sample, and values were normalized to the mock condition at day 1.

### Illustration

Schematic illustration of immunodepletion strategy was generated by modifying images purchased in the PPT Drawing Toolkits—BIOLOGY Bundle from Motifolio, Inc.

### Data presentation and statistical analysis

Graphs are presented as mean ± SEM of 3 or more biological replicates, unless otherwise noted. All statistical analyses were done with 3 or more replicate values per group. Student’s unpaired, two-tailed t tests were used when making comparison between only 2 means. One-way ANOVAs were employed to compare the means of 3 or more groups within one independent variable. Two-way ANOVAs were used for comparisons between two independent variables (e.g. viral treatment vs. antibody treatment). Asterisks for comparisons done by One-Way or Two-Way ANOVA represent the multiplicity-adjusted p values from multiple comparisons tests. Multiple comparisons tests were selected as follows: Dunnett’s post-test for comparing all means to a single control value, Bonferroni’s post-test for comparing only means within one independent variable, and Tukey’s post-test for comparing all means for all independent variables. Choice of post-test was dependent on desired comparisons and is indicated in figure legends. Analyses were done using GraphPad Prism 6.0. Statistical outliers (p < 0.05) were excluded from graphs and analysis using Grubbs’ test on the GraphPad website. For all figures, statistical significance was defined as p < 0.05 (*), but all comparisons were also tested for p < 0.01 (**), p < 0.001 (***), and p < 0.0001 (****). “n.s.” = not significant.

## References

[1] Bhatt S, Gething PW, Brady OJ, Messina JP, Farlow AW, Moyes CL, et al The global distribution and burden of dengue. Nature. 2013;496(7446):504–7. 10.1038/nature12060 23563266 PMC3651993

[2] WHO. Dengue and severe dengue WHO Media Centre2012 [cited 2012 April 10]. Fact sheet N°117]. Available: http://www.who.int/mediacentre/factsheets/fs117/en/.

[3] Guzman MG, Alvarez M, Halstead SB. Secondary infection as a risk factor for dengue hemorrhagic fever/dengue shock syndrome: an historical perspective and role of antibody-dependent enhancement of infection. Arch Virol. 2013;158(7):1445–59. 10.1007/s00705-013-1645-3 . 23471635

[4] Rothman AL. Immunity to dengue virus: a tale of original antigenic sin and tropical cytokine storms. Nat Rev Immunol. 2011;11(8):532–43. doi: nri3014 [pii] 10.1038/nri3014 . 21760609

[5] Murphy BR, Whitehead SS. Immune response to dengue virus and prospects for a vaccine. Annu Rev Immunol. 2011;29:587–619. 10.1146/annurev-immunol-031210-101315 . 21219187

[6] Bhamarapravati N. Hemostatic defects in dengue hemorrhagic fever. Rev Infect Dis. 1989;11 Suppl 4:S826–9. . 2665014 10.1093/clinids/11.supplement_4.s826

[7] Pang T, Cardosa MJ, Guzman MG. Of cascades and perfect storms: the immunopathogenesis of dengue haemorrhagic fever-dengue shock syndrome (DHF/DSS). Immunol Cell Biol. 2007;85(1):43–5. 10.1038/sj.icb.7100008 . 17130899

[8] Halstead SB, O'Rourke EJ. Dengue viruses and mononuclear phagocytes. I. Infection enhancement by non-neutralizing antibody. J Exp Med. 1977;146(1):201–17. 406347 10.1084/jem.146.1.201 PMC2180729

[9] Beltramello M, Williams KL, Simmons CP, Macagno A, Simonelli L, Quyen NT, et al The human immune response to Dengue virus is dominated by highly cross-reactive antibodies endowed with neutralizing and enhancing activity. Cell Host Microbe. 2010;8(3):271–83. doi: S1931-3128(10)00279-9 [pii] 10.1016/j.chom.2010.08.007 . 20833378 PMC3884547

[10] Kou Z, Quinn M, Chen H, Rodrigo WW, Rose RC, Schlesinger JJ, et al Monocytes, but not T or B cells, are the principal target cells for dengue virus (DV) infection among human peripheral blood mononuclear cells. J Med Virol. 2008;80(1):134–46. 10.1002/jmv.21051 . 18041019

[11] Durbin AP, Vargas MJ, Wanionek K, Hammond SN, Gordon A, Rocha C, et al Phenotyping of peripheral blood mononuclear cells during acute dengue illness demonstrates infection and increased activation of monocytes in severe cases compared to classic dengue fever. Virology. 2008;376(2):429–35. doi: S0042-6822(08)00199-2 [pii] 10.1016/j.virol.2008.03.028 18452966 PMC2546568

[12] Bozza FA, Cruz OG, Zagne SM, Azeredo EL, Nogueira RM, Assis EF, et al Multiplex cytokine profile from dengue patients: MIP-1beta and IFN-gamma as predictive factors for severity. BMC Infect Dis. 2008;8:86. doi: 1471-2334-8-86 [pii] 10.1186/1471-2334-8-86 18578883 PMC2474613

[13] Srikiatkhachorn A, Green S. Markers of dengue disease severity. Curr Top Microbiol Immunol. 2010;338:67–82. 10.1007/978-3-642-02215-9_6 . 19802579

[14] Nguyen TH, Lei HY, Nguyen TL, Lin YS, Huang KJ, Le BL, et al Dengue hemorrhagic fever in infants: a study of clinical and cytokine profiles. J Infect Dis. 2004;189(2):221–32. 10.1086/380762 . 14722886

[15] Ubol S, Masrinoul P, Chaijaruwanich J, Kalayanarooj S, Charoensirisuthikul T, Kasisith J. Differences in global gene expression in peripheral blood mononuclear cells indicate a significant role of the innate responses in progression of dengue fever but not dengue hemorrhagic fever. J Infect Dis. 2008;197(10):1459–67. 10.1086/587699 . 18444802

[16] Priyadarshini D, Gadia RR, Tripathy A, Gurukumar KR, Bhagat A, Patwardhan S, et al Clinical findings and pro-inflammatory cytokines in dengue patients in Western India: a facility-based study. PLoS One. 2010;5(1):e8709 10.1371/journal.pone.0008709 20090849 PMC2806829

[17] Rathakrishnan A, Wang SM, Hu Y, Khan AM, Ponnampalavanar S, Lum LC, et al Cytokine expression profile of dengue patients at different phases of illness. PLoS One. 2012;7(12):e52215 10.1371/journal.pone.0052215 23284941 PMC3527385

[18] Jaiyen Y, Masrinoul P, Kalayanarooj S, Pulmanausahakul R, Ubol S. Characteristics of dengue virus-infected peripheral blood mononuclear cell death that correlates with the severity of illness. Microbiol Immunol. 2009;53(8):442–50. doi: MIM148 [pii] 10.1111/j.1348-0421.2009.00148.x . 19659928

[19] Chang DM, Shaio MF. Production of interleukin-1 (IL-1) and IL-1 inhibitor by human monocytes exposed to dengue virus. J Infect Dis. 1994;170(4):811–7. . 7930722 10.1093/infdis/170.4.811

[20] Callaway JB, Smith SA, McKinnon KP, de Silva AM, Crowe JE, Ting JP. Spleen Tyrosine Kinase (Syk) Mediates IL-1β Induction by Primary Human Monocytes During Antibody-Enhanced Dengue Virus Infection. J Biol Chem. 2015 10.1074/jbc.M115.664136 . 26032420 PMC4498069

[21] Goto M, Katayama KI, Shirakawa F, Tanaka I. Involvement of NF-kappaB p50/p65 heterodimer in activation of the human pro-interleukin-1beta gene at two subregions of the upstream enhancer element. Cytokine. 1999;11(1):16–28. doi: S1043-4666(98)90390-8 [pii] 10.1006/cyto.1998.0390 . 10080875

[22] Martinon F, Burns K, Tschopp J. The inflammasome: a molecular platform triggering activation of inflammatory caspases and processing of proIL-beta. Mol Cell. 2002;10(2):417–26. doi: S1097276502005993 [pii]. . 12191486 10.1016/s1097-2765(02)00599-3

[23] Davis BK, Wen H, Ting JP. The inflammasome NLRs in immunity, inflammation, and associated diseases. Annu Rev Immunol. 2011;29:707–35. 10.1146/annurev-immunol-031210-101405 . 21219188 PMC4067317

[24] Wen H, Miao EA, Ting JP. Mechanisms of NOD-like receptor-associated inflammasome activation. Immunity. 2013;39(3):432–41. 10.1016/j.immuni.2013.08.037 24054327 PMC3835203

[25] Guo H, Callaway JB, Ting JP. Inflammasomes: mechanism of action, role in disease, and therapeutics. Nat Med. 2015;21(7):677–87. 10.1038/nm.3893 26121197 PMC4519035

[26] Strowig T, Henao-Mejia J, Elinav E, Flavell R. Inflammasomes in health and disease. Nature. 2012;481(7381):278–86. 10.1038/nature10759 . 22258606

[27] Rathinam VA, Vanaja SK, Fitzgerald KA. Regulation of inflammasome signaling. Nat Immunol. 2012;13(4):333–42. 10.1038/ni.2237 22430786 PMC3523703

[28] Howard AD, Kostura MJ, Thornberry N, Ding GJ, Limjuco G, Weidner J, et al IL-1-converting enzyme requires aspartic acid residues for processing of the IL-1 beta precursor at two distinct sites and does not cleave 31-kDa IL-1 alpha. J Immunol. 1991;147(9):2964–9. . 1919001

[29] Rathinam VA, Vanaja SK, Fitzgerald KA. Regulation of inflammasome signaling. Nat Immunol. 2012;13(4):333–2. doi: ni.2237 [pii] 10.1038/ni.2237 . 22430786 PMC3523703

[30] Poeck H, Ruland J. From virus to inflammation: mechanisms of RIG-I-induced IL-1β production. Eur J Cell Biol. 2012;91(1):59–64. doi: S0171-9335(11)00031-8 [pii] 10.1016/j.ejcb.2011.01.013 . 21481488

[31] Wu MF, Chen ST, Yang AH, Lin WW, Lin YL, Chen NJ, et al CLEC5A is critical for dengue virus-induced inflammasome activation in human macrophages. Blood. 2013;121(1):95–106. 10.1182/blood-2012-05-430090 . 23152543

[32] Smith SA, Zhou Y, Olivarez NP, Broadwater AH, de Silva AM, Crowe JE. Persistence of circulating memory B cell clones with potential for dengue virus disease enhancement for decades following infection. J Virol. 2012;86(5):2665–75. doi: JVI.06335-11 [pii] 10.1128/JVI.06335-11 22171265 PMC3302281

[33] Smith SA, de Alwis AR, Kose N, Jadi RS, de Silva AM, Crowe JE. Isolation of dengue virus-specific memory B cells with live virus antigen from human subjects following natural infection reveals the presence of diverse novel functional groups of antibody clones. J Virol. 2014;88(21):12233–41. 10.1128/JVI.00247-14 25100837 PMC4248927

[34] Sydow FF, Santiago MA, Neves-Souza PC, Cerqueira DI, Gouvea AS, Lavatori MF, et al Comparison of dengue infection in human mononuclear leukocytes with mosquito C6/36 and mammalian Vero cells using flow cytometry to detect virus antigen. Mem Inst Oswaldo Cruz. 2000;95(4):483–9. doi: S0074-02762000000400007 [pii]. . 10904403 10.1590/s0074-02762000000400007

[35] Kang S, Fernandes-Alnemri T, Rogers C, Mayes L, Wang Y, Dillon C, et al Caspase-8 scaffolding function and MLKL regulate NLRP3 inflammasome activation downstream of TLR3. Nat Commun. 2015;6:7515 10.1038/ncomms8515 26104484 PMC4480782

[36] Antonopoulos C, Russo HM, El Sanadi C, Martin BN, Li X, Kaiser WJ, et al Caspase-8 as an Effector and Regulator of NLRP3 Inflammasome Signaling. J Biol Chem. 2015 10.1074/jbc.M115.652321 . 26100631 PMC4536427

[37] Heinz FX, Stiasny K. Flaviviruses and their antigenic structure. J Clin Virol. 2012;55(4):289–95. 10.1016/j.jcv.2012.08.024 . 22999801

[38] Flamand M, Megret F, Mathieu M, Lepault J, Rey FA, Deubel V. Dengue virus type 1 nonstructural glycoprotein NS1 is secreted from mammalian cells as a soluble hexamer in a glycosylation-dependent fashion. J Virol. 1999;73(7):6104–10. 10364366 10.1128/jvi.73.7.6104-6110.1999 PMC112675

[39] Marinho CF, Azeredo EL, Torrentes-Carvalho A, Marins-Dos-Santos A, Kubelka CF, de Souza LJ, et al Down-regulation of complement receptors on the surface of host monocyte even as in vitro complement pathway blocking interferes in dengue infection. PLoS One. 2014;9(7):e102014 10.1371/journal.pone.0102014 25061945 PMC4111305

[40] Uchino S, Bellomo R, Goldsmith D, Davenport P, Cole L, Baldwin I, et al Cytokine removal with a large pore cellulose triacetate filter: an ex vivo study. Int J Artif Organs. 2002;25(1):27–32. . 11853067 10.1177/039139880202500105

[41] Winkelmann ER, Widman DG, Xia J, Ishikawa T, Miller-Kittrell M, Nelson MH, et al Intrinsic adjuvanting of a novel single-cycle flavivirus vaccine in the absence of type I interferon receptor signaling. Vaccine. 2012;30(8):1465–75. doi: S0264-410X(11)02054-8 [pii] 10.1016/j.vaccine.2011.12.103 22226862 PMC3274573

[42] Ader DB, Celluzzi C, Bisbing J, Gilmore L, Gunther V, Peachman KK, et al Modulation of dengue virus infection of dendritic cells by Aedes aegypti saliva. Viral Immunol. 2004;17(2):252–65. 10.1089/0882824041310496 . 15279703

[43] King CA, Anderson R, Marshall JS. Dengue virus selectively induces human mast cell chemokine production. J Virol. 2002;76(16):8408–19. 12134044 10.1128/JVI.76.16.8408-8419.2002 PMC155122

[44] Souza-Neto JA, Sim S, Dimopoulos G. An evolutionary conserved function of the JAK-STAT pathway in anti-dengue defense. Proc Natl Acad Sci U S A. 2009;106(42):17841–6. 10.1073/pnas.0905006106 19805194 PMC2764916

[45] Brown MG, McAlpine SM, Huang YY, Haidl ID, Al-Afif A, Marshall JS, et al RNA sensors enable human mast cell anti-viral chemokine production and IFN-mediated protection in response to antibody-enhanced dengue virus infection. PLoS One. 2012;7(3):e34055 10.1371/journal.pone.0034055 22479521 PMC3316603

[46] Kamau E, Takhampunya R, Li T, Kelly E, Peachman KK, Lynch JA, et al Dengue virus infection promotes translocation of high mobility group box 1 protein from the nucleus to the cytosol in dendritic cells, upregulates cytokine production and modulates virus replication. J Gen Virol. 2009;90(Pt 8):1827–35. 10.1099/vir.0.009027-0 . 19369409

[47] Shafee N, AbuBakar S. Characterization of dengue type 2 NGC virus infection in C6/36, Vero and MRC-5 cells. International Journal of Virology. 2011;7(1):24–32.

[48] Teshima T, Harada M, Takamatsu Y, Makino K, Taniguchi S, Inaba S, et al Cytotoxic drug and cytotoxic drug/G-CSF mobilization of peripheral blood stem cells and their use for autografting. Bone Marrow Transplant. 1992;10(3):215–20. . 1384898

[49] Widman DG, Ishikawa T, Fayzulin R, Bourne N, Mason PW. Construction and characterization of a second-generation pseudoinfectious West Nile virus vaccine propagated using a new cultivation system. Vaccine. 2008;26(22):2762–71. doi: S0264-410X(08)00309-5 [pii] 10.1016/j.vaccine.2008.03.009 . 18423946

[50] Gurukumar KR, Priyadarshini D, Patil JA, Bhagat A, Singh A, Shah PS, et al Development of real time PCR for detection and quantitation of Dengue Viruses. Virol J. 2009;6:10. doi: 1743-422X-6-10 [pii] 10.1186/1743-422X-6-10 19166574 PMC2651855

[51] Wu C, Orozco C, Boyer J, Leglise M, Goodale J, Batalov S, et al BioGPS: an extensible and customizable portal for querying and organizing gene annotation resources. Genome Biol. 2009;10(11):R130 10.1186/gb-2009-10-11-r130 19919682 PMC3091323

[52] Su AI, Wiltshire T, Batalov S, Lapp H, Ching KA, Block D, et al A gene atlas of the mouse and human protein-encoding transcriptomes. Proc Natl Acad Sci U S A. 2004;101(16):6062–7. 10.1073/pnas.0400782101 15075390 PMC395923

